# Childhood trajectories of internalising and externalising problems associated with a polygenic risk score for neuroticism in a UK birth cohort study

**DOI:** 10.1002/jcv2.12141

**Published:** 2023-02-22

**Authors:** Ilaria Costantini, Hannah Sallis, Kate Tilling, Daniel Major‐Smith, Rebecca M. Pearson, Daphne‐Zacharenia Kounali

**Affiliations:** ^1^ Centre for Academic Mental Health University of Bristol Bristol UK; ^2^ Department of Population Health Sciences, Bristol Medical School University of Bristol Bristol UK; ^3^ Medical Research Council (MRC) Integrative Epidemiology Unit University of Bristol Bristol UK; ^4^ School of Psychological Science University of Bristol Bristol UK; ^5^ Centre for Academic Child Health Bristol Medical School University of Bristol Bristol UK; ^6^ Department of Psychology Manchester Metropolitan University Manchester UK

**Keywords:** ALSPAC, childhood internalising and externalising trajectories, neuroticism, polygenic risk score

## Abstract

**Background:**

Neuroticism represents a personality disposition towards experiencing negative emotions more frequently and intensely. Longitudinal studies suggest that neuroticism increases risk of several psychological problems. Improved understanding of how this trait manifests in early life could help inform preventative strategies in those liable to neuroticism.

**Methods:**

This study explored how a polygenic risk score for neuroticism (NEU PRS) is expressed from infancy to late childhood across various psychological outcomes using multivariable linear and ordinal regression models. In addition, we employed a three‐level mixed‐effect model to characterise child internalising and externalising trajectories and estimate how a child PRS associated with both their overall levels and rates of change in 5279 children aged 3–11 in the Avon Longitudinal Study of Parents and Children cohort.

**Results:**

We found evidence that the NEU PRS was associated with a more emotionally sensitive temperament in early infancy in addition to higher emotional and behavioural problems and a higher risk of meeting diagnostic criteria for a variety of clinical disorders, particularly anxiety disorders, in childhood. The NEU PRS was associated with overall levels of internalising and externalising trajectories, with a larger magnitude of association on the internalising trajectory. The PRS was also associated with slower rates of reduction of internalising problems across childhood.

**Conclusions:**

Our findings using a large, well‐characterised birth cohort study suggest that phenotypic manifestations of a PRS for adult neuroticism can be detected as early as in infancy and that this PRS associates with several mental health problems and differences in emotional trajectories across childhood.

## Graphical abstract

We examined the association between a polygenic risk score for neuroticism and a host of psychological outcomes in addition to its association with trajectories of emotional and behavioural problems in the Children of the 90s. We found that the PRS associated with a more difficult temperament, more emotional and behavioural problems, and greater risk of being diagnosed with a clinical disorder in childhood. The PRS was also associated with higher levels of emotional and behavioural problems trajectories and dampened recovery from emotional problems across childhood.

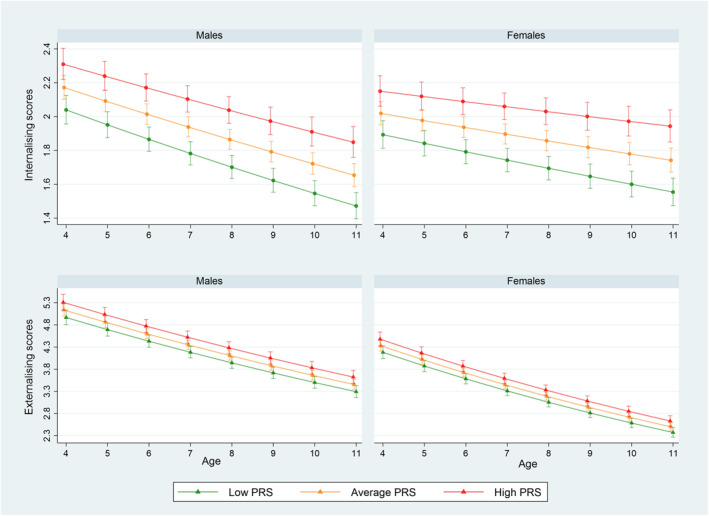

**Key points**

- Neuroticism is an important risk factor for mental health problems with a moderate heritable component; however, it is unclear how genetic liability to neuroticism associates with severity and persistence of emotional and behavioural problems across childhood.
- The present study used a prospective design to examine the association between a polygenic risk score for neuroticism and a host of psychological outcomes in addition to its association with trajectories of emotional and behavioural problems in over 6000 children.
- We found that the PRS associated with a more difficult temperament, more emotional and behavioural problems, and greater risk of being diagnosed with a clinical disorder in childhood. The PRS was also associated with higher levels of emotional and behavioural problems trajectories and dampened recovery from emotional problems across childhood.
- Our study illustrates the importance of genetic in the severity and persistence of emotional problems across childhood. Trio analyses may elucidate the causal relationship between genetic liability to neuroticism and child psychiatric outcomes.

## INTRODUCTION

Neuroticism refers to a personality trait characterised by an individual's tendency to experience negative emotions more frequently and intensely (Eysenck, 1991). Longitudinal studies suggest that neuroticism is a risk factor for a variety of psychological and physical problems such as anxiety, depression, adverse cardiovascular events, and substance misuse (Khan et al., 2005; Malouff et al., 2005; Saulsman & Page, 2004). Consequently, this personality trait could represent an important personal, economical, and societal burden (Lahey, 2009). The relationship between neuroticism and mental health problems is complex and there are likely multiple pathways linking this personality disposition to later mental health issues. Recent evidence has suggested that a general neuroticism factor overlaps substantially with both internalising (e.g., anxiety and depression symptoms) and externalising (e.g., hyperactivity and conduct problems) problems in childhood and that shifting the target of intervention to the development of emotional coping strategies and resilience in children may be more effective in preventing later onset of mental and physical health problems in those with this trait (Barlow et al., 2014; Brandes et al., 2019).

Twin and family‐DNA informed studies (Boomsma et al., 2018; Cheesman et al., 2020) have suggested a heritability of neuroticism of approximately 30%, indicating a moderate genetic contribution to this trait. In addition, genome‐wide association studies (GWASs) have identified hundreds of independent loci contributing to its aetiology (Luciano et al., 2018; Nagel et al., 2018). Thus, it is increasingly possible to construct polygenic risk scores (PRS) as indicators of an individual's genetic liability for neuroticism (Lewis & Vassos, 2020) in populations of European ancestry (Rosenberg et al., 2010).

Some longitudinal studies have explored phenotypic manifestations of PRSs for various psychiatric disorders (e.g., schizophrenia—SCZ [Jansen et al., 2018; Jones et al., 2016]), and childhood psychopathology (e.g., ADHD [Stergiakouli et al., 2017]). Furthermore, a recent study using ALSPAC has explored the association between seven PRSs (including SCZ and major depressive disorder, MDD) and internalising and externalising developmental trajectories (Speyer, Neaves, et al., 2022). Interestingly, SCZ and MDD PRSs did not discriminate individuals belonging to different internalising and externalising classes. More recently, few studies employing either twin study design or longitudinal cohort studies have used a PRS for neuroticism (henceforth NEU PRS) as derived in adulthood and explored its association with a variety of outcomes in childhood and adolescence (Chen et al., 2022; Neumann et al., 2021). However, no studies to date have evaluated the association of a NEU PRS with trajectories of emotional and behavioural problems across any age group (see Akingbuwa et al., 2020 for a meta‐analysis of these studies) and explored its phenotypic manifestation early in infancy. Exploring phenotypic manifestations of a genetic predisposition to psychological traits like neuroticism in infancy and childhood and its association with the development and maintenance of trajectories of psychological problems could help in understanding how genetic liability to neuroticism influence the onset of internalising and externalising problems. This may be particularly important in the context of child psychiatry where psychological symptoms are not as differentiated as in later ages (e.g., middle to late adolescence) and were historically understudied and their prevalence underappreciated (Ramchandani, 2004; Skovgaard et al., 2004; Tandon et al., 2009).

Thus, the primary aims of this study were to examine phenotypic manifestations of a NEU PRS across a host of temperamental traits, psychological problems, and clinical disorders in infancy and childhood. Second, we aimed to examine how a NEU PRS associates with childhood trajectories of internalising and externalising problems where repeated measures were available.

## MATERIALS AND METHODS

### Participants

The Avon Longitudinal Study of Parents and Children (ALSPAC), also known as Children of the 90s, is an ongoing prospective population‐based birth cohort study. Between 1990 and 1992, 14,541 pregnant women were recruited in Bristol and the surrounding area, previously known as Avon County. The original mothers and partners (Generation 0: ALSPAC‐G0) (Fraser et al., 2013) and their living children (Generation 1: ALSPAC‐G1) (Boyd et al., 2013) have been followed‐up regularly since recruitment through questionnaires and clinic assessments. When the oldest children were approximately 7 years of age, an attempt was made to bolster the initial sample with eligible cases who had failed to join the study originally, resulting in an additional 913 children being enroled. The total sample size for analyses using any data collected after the age of seven is therefore 15,454 pregnancies, resulting in 15,589 foetuses. Of these 14,901 were alive at 1 year of age (Northstone et al., 2019). This study focuses on the index children (ALSPAC‐G1) and on how their NEU PRS is expressed across childhood. Please note that the study website contains details of all the data that is available through a fully searchable data dictionary and variable search tool http://www.bristol.ac.uk/alspac/researchers/our‐data/. Ethical approval for the study was obtained from the ALSPAC Law and Ethics Committee and South‐West National Health Service (NHS) Research Ethics Committee; informed consent for the use of data collected via questionnaires and clinics was obtained from participants following the recommendations of the ALSPAC Ethics and Law Committee at the time. Consent for biological samples has been collected in accordance with the Human Tissue Act (2004).

### Genetic data

Genotyped data were available on 7851 children and 7826 mothers in the ALSPAC study. Details of genotyping and quality control measures are available in Appendix S1. PRSs for genetic liability for neuroticism were derived for mothers and children and standardised prior to analysis. These scores were based on publicly available summary statistics from a recent GWAS of neuroticism (*N* = 329,821) which identified 116 independent genetic variants at a genome‐wide significance threshold (*p* < 5 × 10^−8^) (Luciano et al., 2018).

PRS were calculated using PRSice‐2 (Choi & O’Reilly, 2019) and restricted to SNPs with a minor allele frequency of >1% and an info score of >0.8. Scores were created by summing the number of risk alleles present for each SNP (0, 1, or 2) weighted by the effect estimates from the original GWAS. Thirteen PRS were derived using *p*‐value thresholds ranging from *p* < 0.5 to *p* < 5 × 10^−8^ (Table S1), with a greater number of SNPs included as the *p*‐value thresholds became less conservative. Our primary analysis used a PRS based on a threshold of *p* < 0.05, which is the threshold that most optimally captured the trade‐off between variance explained for neuroticism liability (maximum amount of variance, Nagelkerke's *R* ^2^, explained in neuroticism) and added error term (Luciano et al., 2018). Using this *p*‐value threshold, the neuroticism polygenic score explained 2.79% of the variance in neuroticism (*β* = 0.19, *p* = 2.65 × 10^−47^) (Luciano et al., 2018).

Child and mother NEU PRSs were standardised using *z*‐scores to have mean = 0 and SD = 1, with a higher PRS indicating a higher liability to neuroticism.

### Outcome measures

Further details for all the outcome measures are reported in Appendix S2 and in Tables S2 and S3. An illustration of the measures used, the timings at which they were measured, and reporters is provided in Figure S1.

### Carey Infant Temperament Scales (CTSs)—6 and 24 months

Carey Infant Temperament Scales (CTSs) (Carey & McDevitt, 1977, 1978) is a common questionnaire used to assess child's temperament (examples of items: ‘Child cries when left playing alone,’ ‘child plays quietly with toys’) which comprises a number of age‐appropriate questions relating to different temperament domains. In this analysis, we used the prorated score which was corrected for age at completion of the questionnaire and gestation at delivery. The results are coded so that higher scores indicate a more ‘difficult’ temperament.

### Strengths and Difficulties Questionnaire (SDQ)—4 to 11 years

The Strengths and Difficulties Questionnaire (SDQ) (Goodman, 1997) is a brief behavioural screening questionnaire validated for children who are approximately 3–16 years old. It provides a score ranging from 0 to 40, where higher scores indicate a larger number of emotional and behavioural difficulties exhibited by the child.

### Other psychological outcomes—8 years

Children's locus of control, self‐esteem, and IQ were assessed using validated scales.

### Axis‐I disorders—7 and 10 years

Any axis‐I disorders (i.e., clinical conditions such as anxiety and depressive disorders) classified using the Diagnostic and Statistical Manual of Mental Disorders (DSM‐IV) (Bell, 1994) (yes vs. no) and the International Statistical Classification of Diseases and Related Health Problems, Tenth Revision (ICD‐10) (Brämer, 1988) were assessed using maternal and a mix of maternal and teacher reports from the Development and Well‐being Assessment (DAWBA) (Goodman, Heiervang, et al., 2011; R. Goodman et al., 2000) when children were 7 and 10 years old. Up to six ordinal bands were derived with a computer algorithm which has been shown to be similar in accuracy to a clinical rating method (Goodman, Heiervang, et al., 2011). Each band corresponds to the approximate prevalence of such disorders in an epidemiological sample, with 0 representing the lowest prevalence and 6 the highest. Higher scores indicate a higher probability of being diagnosed with such disorders.

### Negative and positive control variables

Negative and positive controls are usually employed in epidemiological analyses to test the validity of the association examined (Lipsitch et al., 2010). The emotional stability subscale (i.e., inverse of neuroticism) of the Big Five personality test (Goldberg, 1992) and variables capturing whether the child wore glasses or not were used as outcomes in positive and negative control analyses, respectively.

### Covariate measures

In all our models we included age of the child, sex of the child, and the first five principal components of genetic ancestry (PCs) as covariates. Adjusting for principal components of ancestry is a standard approach to account for population stratification (e.g., due to differences in ethnicity and/or geographical location) in genetic analyses (Gaspar & Breen, 2019; McVean, 2009). We used twenty PCs as post‐hoc analyses but, as they did not change the results, we report here only the results obtained using five PCs, which are those that accounted for the largest variation in a relatively homogenous sample such ALSPAC. In addition, to support the missing at random (MAR) assumption of the mixed effect models, we included variables indicating maternal education, maternal social class, and maternal age as they were strongly associated with the probability of having missing data.

### Statistical analyses

All analyses were conducted using Stata 16 statistical software 2019. Betas (*β*) indicate a unit difference increase or reduction in the outcome score for each 1SD increase in the exposure.

### Cross‐sectional multivariable regression models

To examine the phenotypic manifestation of a NEU PRS across childhood, we used separate multivariable linear regression models with robust standard errors (SEs) to estimate coefficients and 95% CIs for the associations between the child NEU PRS and different continuous psychological outcomes (i.e., CTSs, SDQ, Locus of control, Self‐Esteem, and IQ) and ordinal logistic regressions for discrete ordinal outcomes (i.e., DAWBA), adjusted for the above‐mentioned covariates. Using robust SEs (i.e., Huber‐White Sandwich estimators) can help in addressing potential violations of linear regression assumptions such as normality, heteroscedasticity, or observations that exhibit large residuals. Primary analyses were conducted on the complete case dataset. As a positive and negative control analyses respectively, we investigated whether the child PRS for neuroticism was associated with the neuroticism (i.e., inverse of emotional stability) subscale as measured by the Big Five at 13 years of age and with the child wearing glasses at 8 and 10 years of age.

To take into account a potential inflated false discovery rate due to multiple testing introduced by using separate models for different time‐points and various subscales, a heuristic Bonferroni adjusted *p*‐value threshold was set at *p* < 0.0014 (0.05/35, where 35 pertains to each unique outcome tested). However, as we have included subscales which are highly correlated with each other, this threshold is likely to be overly conservative. We do not present Bonferroni‐adjusted *p*‐values as we discourage a binary interpretation of evidence using the arbitrary cut‐off of 0.05 (Sterne & Smith, 2001). Thus, we discuss the strength of the evidence as being on a continuum from ‘very strong evidence’ (against the null) and ‘weak evidence’ (against the null). We also try to include more factors other than the *p*‐value in interpretation of the statistical evidence for our findings (e.g., the plausible range and potential importance of the values covered by the 95% CIs, the number of tests performed, the robustness to sensitivity analyses, and prior beliefs in the truthfulness of the results).

### Linear mixed effect model for the characterisation of trajectories of internalising and externalising problems

**FIGURE 1:**
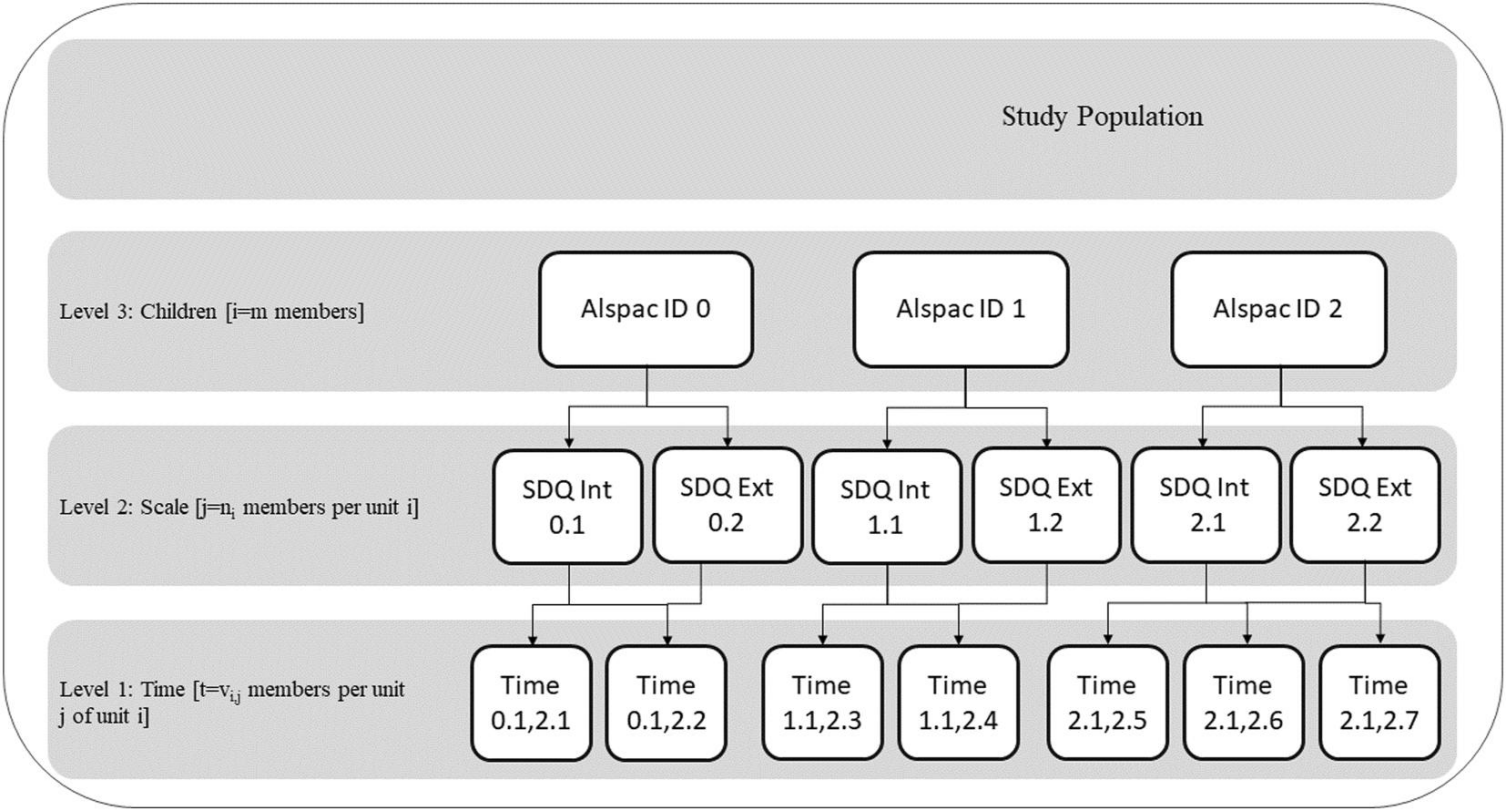
The figure illustrates the hierarchical structure of the selected linear mixed‐effect model.

In this study, a three‐level mixed‐effect linear model (command *mixed* in Stata‐16) with random intercepts and slopes was employed to characterise behavioural and emotional trajectories over time and to examine the association of the child PRS with the overall score of emotional and behavioural problems and how these change according to sex, type of scale, and reporter of the scale. The levels represent a dependence structure where children's scores at different measurement occasions (level 1) are nested within the type of scale (internalising or externalising) (level 2) which are nested within an individual child (level 3) (Figure 1) (Naumova et al., 2001).

This model allows a separate intercept and slope for each child in each scale and it permits the investigation of the effect of covariates as fixed effects. The model included a random intercept and slopes for the change over time which permitted estimation of individual child variation in score levels, and how these change with time across different scales and reporters. Restricted maximum likelihood (REML) was employed instead of maximum likelihood (ML) for fitting this model. Model adequacy was assessed using information criteria (i.e., AIC and BIC) and examining model assumptions, namely homogeneity of variance (with group variance being constant between groups and predictors), linearity, and normality of residual variances at different levels. This model was selected among five different models (4 hypothesised a priori and 1 tested post‐hoc).

The sample used in three‐level linear mixed‐models consisted of 5729 children with at least one observation per internalising and externalising subscale over a maximum of seven occasions per scale. The mean number of measurements was 5.62 (SD = 3.50). The majority (74.5%) of the child questionnaires on the SDQ for this study were answered by the mother for the parent reported scale. To examine whether the child PRS for neuroticism was associated with the overall level of the trajectories, we included the PRS as a fixed‐effect term and we used interaction terms to explore its effects depending on the scale used, the reporter of the scale, and the sex of the child. To examine how the PRS was associated with changes over time in our model, we included a main effect of the PRS and an interaction of the PRS with the fixed‐effect age term.

### Sensitivity analyses

We employed various sensitivity analyses to test the potential presence of differential and dependent measurement error due to the maternal PRS, confounding via maternal genotype, genetic pleiotropy, and bias arising from levels of missingness. For more details about the sensitivity analyses refer to Appendix S3.

## RESULTS

### Descriptive results

The numbers of individuals who participated in the different assessments at various visits are shown in Tables S2 and S3. Of the 7851 ALSPAC children with genetic data available (51.25% male and 48.75% female), 6271 to 3579 participated in assessments from 6 months to 11 years of age, respectively. Of the participants who had genetic information, missingness for the outcomes of interest was modest at 6 months of age (20.42% missingness) and high when the SDQ was measured by the teacher at age 8 (up to 54.50% missingness) (see Figure S3 for a Flow diagram of ALSPAC participants). Participants who had data on the variables included in the different models (e.g., outcome, exposure, and covariates) generally had mothers who were of a higher social class, obtained a higher education, and who were less likely to have smoked during pregnancy (Table S4). Correlations between subscales of SDQ and between mother and teacher reports are reported in Appendix S4 and Table S5.

### Cross‐sectional analyses of the association between child NEU PRS and various psychological outcomes

Both negative and positive control analyses supported the validity of our analyses (see ‘Positive and negative control analyses’ in Appendix S4).

**FIGURE 2:**
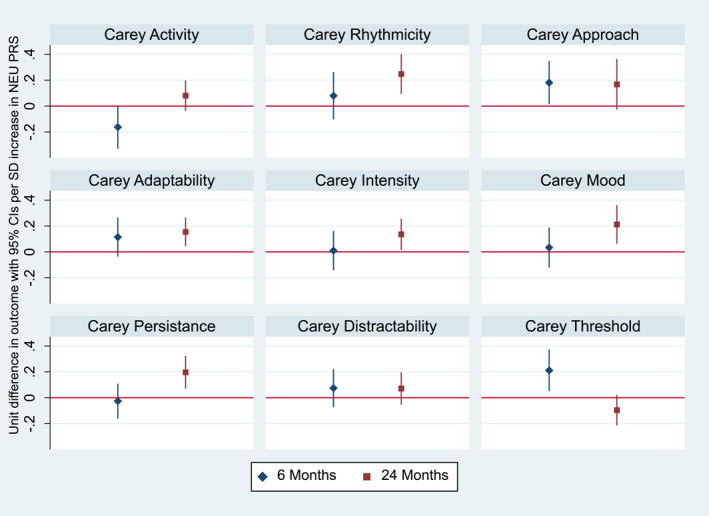
The figure represents the cross‐sectional associations obtained using linear regressions between a child NEU PRS and CTS at 6 and 24 months.

We found some suggestive evidence of an association between child NEU PRS and more difficult temperament at 6 months. Both the strength of the evidence and the size of the association strengthen at 24 months. However, evidence to support some of these associations weakened when considering a heuristic *p*‐value threshold for multiple testing (complete findings are presented in Table S6 and Figure 2).

**FIGURE 3:**
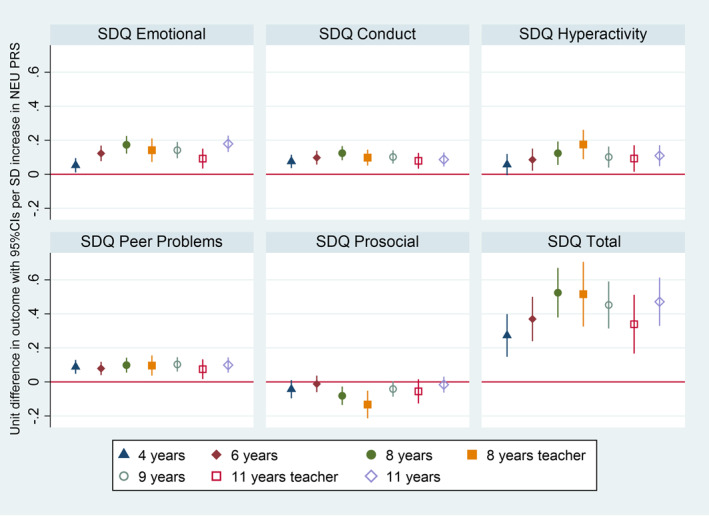
The figure represents the cross‐sectional associations obtained using linear regressions between a child NEU PRS and SDQ from 3 to 11 years of age.

We also found supporting evidence for an association between child NEU PRS and all the subscales of the SDQ but the prosocial scale. In particular, there was strong evidence that a higher neuroticism PRS was associated with higher scores in both the emotional and behavioural domains of the SDQ (i.e., total score) across all seven occasions from 4 to 11 years of age. Effect sizes generally increased throughout childhood with the smallest estimates at age 4 (*β* = 0.27, 95% CI: 0.15–0.40, *p* = 2.30 × 10^−5^) and the largest at age 8, both when reported by the mother (*β* = 0.52, 95% CI: 0.38–0.67, *p* = 2.08 × 10^−12^) and when reported by the teacher (*β* = 0.52, 95% CI: 0.33–0.71, *p* = 8.13 × 10^−8^). These findings were consistent independent of whether the outcome was teacher or parent reported (Table S6 and Figure 3).

**FIGURE 4:**
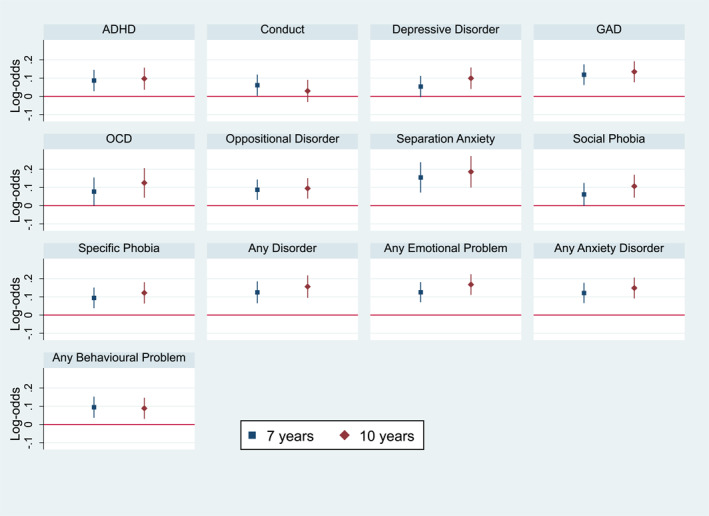
The figure represents the cross‐sectional associations obtained using ordinal logistic regressions between a child NEU PRS and DAWBA at 7 and 10 years of age.

In addition, we observed strong evidence of an association between the neuroticism PRS and the likelihood of receiving clinical diagnoses at both 7 and 10 years of age as measured with the DAWBA. A higher NEU PRS was associated with a 13% (95% CI: 6%–20%) increased risk of generalised anxiety disorder (GAD) and a 14% (95% CI: 8%–21%) increased risk at 7 and 10 years of age, respectively. Similarly, we found strong evidence that children with a higher NEU PRS were at 13% (95% CI: 7%–20%) and 19% (95% CI: 12%–26%, *p* = 2.80 × 10^−8^) increased risk of any anxiety related disorder at 7 and 10 years of age, respectively. When exploring the association between the NEU PRS and any behavioural disorder as measured as a combined score across three traits (i.e., conduct disorder, oppositional defiant disorder, and ADHD), we found nominal evidence that the PRS was associated with a 9% (95% CI: 4%–16%) and 9% (95% CI: 3%–16%) increased risk of this combined score at 7 and 10 years of age, respectively. We found little evidence of an association between the child NEU PRS and depressive disorders at 7 years of age (OR = 1.05, 95% CI: 0.99–1.12), but stronger evidence of an association at 10 years of age (OR = 1.11, 95% CI: 1.04–1.17) (Table S7 and Figure 4).

Overall, child NEU PRS seems to be manifest with some specificity for anxiety related problems, already in early childhood. Finally, we also observed strong evidence that a higher NEU PRS was positively associated with broader psychological constructs such as having an external locus of control and inversely associated with global and scholastic self‐esteem and IQ (Table S6).

### Child NEU PRS and trajectories of internalising and externalising problems

We estimated five main models (see Appendix S5 for more details and Table S8 for model fit). Here we present the selected model.

The overall mean for the internalising scale of the SDQ was estimated as 2.97 (95% CI: 2.67–3.35) for boys when assessed by the parents. For every SD increase in child NEU PRS, we observed an average 6% (95% CI: 4%–7%) increase in internalising problems in boys as reported by the parent. The magnitude of this effect was halved (i.e., 3%, 95% CI: 2%–5%) when examining externalising problems. We also observed strong evidence of a rate of reduction of 3% (95% CI: 2%–3%) per year of age on the internalising scores in boys when reported by the parents, which was larger than the rate of reduction in girls by 1% (95% CI: 1%–2%). This rate of reduction was more pronounced on the externalising scale (rising to 6% per year, 95% CI: 4%–6%). The PRS effect on the rate of change with age became more pronounced on the internalising scale where the rates of change were slower (given by the three‐way interaction between NEU PRS, age, and scale: −0.003%; 95% CI: −0.01% to −0.0005%). We observed similar patterns of the effect of the PRS in girls. Differences between sexes mainly consisted of differences in score levels with age according to scale and reporter. Such findings are described by the three‐way interactions between reporters, scale, and sex. For example, we found that teachers scored girls 17% (95% CI: 11%–24%) lower on the externalising scale than parents but the difference between reporters is smaller than when rating boys. Finally, we found that teachers gave lower scores to girls with age on the internalising scale (reduction of 4% with each year of age, 95% CI: 2%–5%). Further discussion of findings from this model are reported in the Appendix S5. Complete findings from both the fixed and random effects parts of the selected model are presented in Table 1. Mean differences on the actual scale scores between low and high PRS at the youngest and oldest ages are reported in Table S9.

**TABLE 1:** Findings for the three‐level model investigating the association between PRS and SDQ across time.

Fixed‐effect parameters	Value of category	Log coefficients (95% CIs)^a^
Scale	Internalising = 0, Externalising = 1	0.58 (0.56 to 0.60)
Time‐level variables		
Age	Age in years, centred at 8 years	−0.03 (−0.03 to −0.02)
Child‐level variables		
Sex	Males = 0, Females = 1	−0.003 (−0.033 to 0.030)
PRS neuroticism	*z*‐scores	0.06 (0.04 to 0.07)
Reporter	Parent = 0, Teacher = 1	−0.11 (−0.14 to −0.07)
PRS ∗ reporter		−0.0005 (−0.023 to 0.024)
PRS ∗ scale		−0.03 (−0.04 to −0.01)
PRS ∗ age		0.004 (0.001 to 0.007)
PRS ∗ age ∗ scale		−0.003 (−0.01 to −0.0005)
PRS ∗ scale ∗ reporter		0.03 (−0.01 to 0.06)
PRS ∗ reporter ∗ age		−0.2 (−0.02 to −0.01)
Reporter ∗ sex		−0.12 (−0.17 to −0.07)
Reporter ∗ scale		−0.24 (−0.28 to −0.19)
Reporter ∗ scale ∗ sex		−0.17 (−0.24 to −0.11)
Reporter ∗ age		0.03 (0.02 to 0.04)
Sex ∗ age		0.01 (0.01 to 0.02)
Reporter ∗ sex ∗ age		−0.04 (−0.05 to −0.02)
Scale ∗ age		−0.02 (−0.025 to −0.020)
Scale ∗ sex		−0.20 (−0.23 to −0.16)
Scale ∗ age ∗ sex		−0.03 (−0.03 to −0.02)
Maternal education	1 = low education, 2 = average education, 3 = high education	−0.04 (−0.05 to −0.02)
Maternal social class	0 = low, 1 = high	0.04 (0.01 to 0.06)
Mother's age		−0.01 (−0.012 to −0.01)
1st PC		0.44 (−0.66 to 1–54)
2nd PC		−2.17 (−3.94 to −0.40)
3rd PC		0.38 (−0.68 to 1.43)
4th PC		0.16 (−0.90 to 1.21)
5th PC		−0.35 (−1.39 to 0.69)
Intercept		1.38 (1.30 to 1.47)

Random‐effects parameters	Variance components (95% CIs)
Unstructured individual child level	
Sd (age_8y)	0.05 (0.04 to 0.05)
Sd (_cons)	0.32 (0.31 to 0.33)
Corr (age_8y, cons)	0.57 (0.51 to 0.62)
Unstructured scale‐level	
Sd (reporter)	0.56 (0.55 to 0.58)
Sd (_cons)	0.38 (0.37 to 0.39)
Corr (reporter, cons)	−0.47 (−0.50 to −0.44)
Sd (residual)	0.46 (0.45 to 0.46)
AIC^b^	102,130.7
Log‐likelihood	−51029.34

*Notes*: Reporter: 0 indicates parents and 1 indicates teachers; sex: 0 indicates boys and 1 indicates girls; scale: 0 indicates internalising and 1 indicates externalising; maternal education: 1 indicates low education (equivalent to O level, Certificate of Secondary Education [CSE] or vocational training), 2 indicates A level, and 3 indicates some degree; Mother’s age: Maternal age in years during pregnancy; pc1_child‐pc5_child represents the list of the first five principal components of genetic ancestry.

^a^ Outcome is transformed into the log‐scale and added 1: ln (*x* +1). The coefficients (apart from the intercept) represent the percentage change in the outcome. By exponentiating the intercept and removing 1 (exp(*x*)−1), the intercept indicates the mean score on the actual internalising subscale at 8 years of age in boys and when reported by the parent.

^b^ Akaike Information Criterion.

**FIGURE 5:**
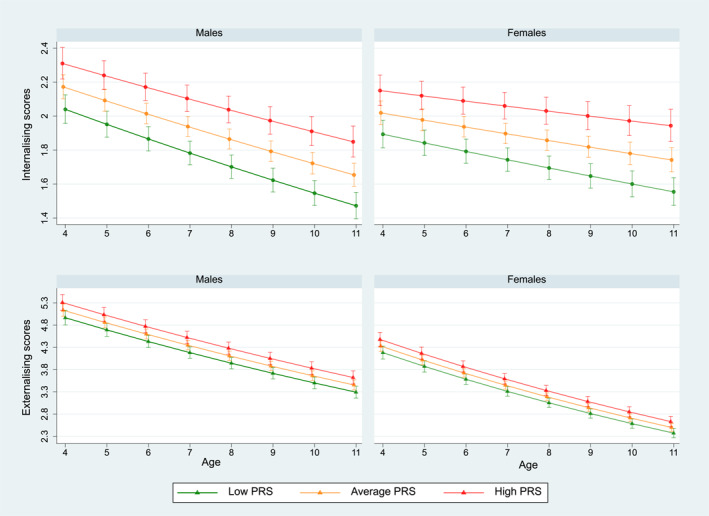
The figure illustrates the trajectories of internalising and externalising problems from 3 to 11 years of age obtained using a three‐level mixed‐effect model. PRS levels are shown and the graphs are separated by sex of the child. Scores were obtained using the SDQ as reported by the mother of the child.

A graphical representation of these marginal model predictions according to age, sex, PRS levels, and reporter is presented in Figure 5 and Figure S6.

### Sensitivity analyses

Sensitivity analyses testing potential differential misclassification of the outcome and bias arising from attrition largely confirmed our main analyses suggesting that such biases, if present, may be negligible in our sample (see Appendix S6 and S7, respectively for more details). In addition, adjusting for maternal NEU PRS to estimate the potential causal association between child NEU PRS and later psychological problems led to a notable reduction in precision (wider CIs) but consistent effect estimates (see Appendix S8 for more details). Finally, sensitivity analyses testing the specificity of the association of neuroticism and our traits of interest (by using a genome wide significant *p*‐value threshold) found attenuated associations across all SDQ scales with the exception of the emotional subscale, suggesting that the observed association between child NEU PRS and the conduct and behavioural subscales may be driven by underlying symptoms of anxiety (see Appendix S9 for more details). All sensitivity analyses results are reported in Tables S10–S16.

## DISCUSSION

Psychiatric genetic studies have recently started investigating associations between genetic variants linked to psychological and psychiatric outcomes in adults and their correlates in children and adolescents (Akingbuwa et al., 2020, 2021).

In this prospective cohort study, we examined multiple cross‐sectional analyses and a three‐level mixed‐effect model to explore the associations between child NEU PRS and a host of behavioural and emotional problems across childhood. In cross‐sectional analyses, we found suggestive evidence of an association with early temperament measures, at 6 and 24 months of age. Child NEU PRS was also found to be associated with both emotional and behavioural problems as well as clinical disorders across childhood. However, the largest effect sizes were found for internalising problems and anxiety‐related clinical disorders as measured by the DAWBA, indicating some specificity in the phenotypical expression of the NEU PRS.

In addition, when estimating trajectories of child internalising and externalising problems between 4 and 11 years of age, we found that they both reduced with age, in line with early childhood emotional issues (i.e., terrible twos) often regulating by late childhood (Paul et al., 2021). Both girls and boys had higher overall levels of externalising, as compared to internalising problems; however, the reduction of externalising trajectories over time was sharper than for internalising trajectories. A higher NEU PRS was associated with an increase in the overall level of internalising problems, with the magnitude of effect halved for the externalising scale. We also found that a higher NEU PRS was associated with smaller rates of reduction of internalising problems, suggesting a dampened recovery from the emotional problems experienced in children with higher levels of this PRS (Figure 5). Overall levels and the rate of decrease of the trajectories were also influenced by the reporter of the scale: with the teacher reporting smaller rates of reduction compared to parents (Appendix S5 and S3). However, both parents and teachers scored girls lower on externalising problems and higher on internalising problems as compared to boys. These findings are consistent with psychological and psychiatric literature which has identified higher levels of total difficulties across childhood in boys as compared to girls (Clark et al., 2016; Gartstein et al., 2009) and literature on teachers reporting fewer problems than parents (Gartstein et al., 2009; Youngstrom et al., 2000). Finally, we found strong evidence of an association between the child NEU PRS and other psychological traits (e.g., locus of control and self‐esteem) and IQ. As IQ and self‐esteem were measured at the same age, it is difficult to speculate about the directionality of the association. For example, the association between NEU PRS and lower IQ could be partially mediated through low scholastic self‐esteem or the other way around (i.e., an association between the PRS and low scholastic self‐esteem is mediated by performance on IQ tests).

Our findings suggest that a child NEU PRS show patterns of specificity that influence both the severity and rate of change in internalising problems throughout childhood and then into early adolescence. NEU PRS seems to be influencing not only overall levels of emotional problems but also their persistence in childhood.

Whilst adjusting for maternal PRS (i.e., a potential confounder of these associations) led to a reduction of the strength of the evidence across all findings, the effects sizes remained comparable with the unadjusted complete case analyses. Findings on internalising domain remained robust to this adjustment. Conversely, we found sharp reductions in all our effect estimates when a PRS at a genome‐wide significance threshold; with little evidence of association remaining for the emotional problems as identified by the SDQ and the anxiety‐specific related problems as identified by the DAWBA. These findings suggest that our results are unlikely to be entirely mediated via genetic liability to neuroticism but likely reflect the effect of pleiotropic pathways from the neuroticism PRS to outcomes examined.

### Strengths and limitations

This study has several strengths. First, we used a large, well‐characterised, prospective population‐based sample to examine neuroticism‐related phenotypes during childhood, a key period of development that closely predates the start of the sharp rise in incidence of internalising problems (i.e., early adolescence) (Natsuaki et al., 2009). Second, the use of repeated measures and the availability of different reporters can provide greater insights in how NEU PRS influence the onset and persistence of child internalising and externalising problems and shed light onto possible misclassification of the outcomes. Employing a three‐level mixed‐effect model offers multiple advantages compared to single‐level analyses. First, such models appropriately account for the dependence found in correlated data. Second, they can also address more complex questions using a single statistical model, thus reducing statistical testing, and the risk of Type I errors. For example, we were able to make a direct comparison between the rate of symptoms change with age across scales (internalising vs. externalising) thus assessing a cross‐level interaction, and to estimate the variance components within groups using ICC. Third, as recommended in Tubbs et al. (2020), when trying to estimate the causal association between the child PRS and an outcome that could be confounded by parental genetics, we adjusted for at least one of the available PRSs (in our case, the maternal NEU PRS). However, it is important to emphasise that if paternal genotype confounds this association, the child estimate will be biased towards the paternal effect, albeit to a lesser extent that if maternal genotype were not adjusted for. Fourth, our results remained robust to a variety of sensitivity analyses testing attrition and potential differential misclassification. In fact, previous studies support the existence of a ‘distortion model’ (Fergusson et al., 1993; Müller et al., 2011; Vierhaus & Lohaus, 2008), for which reporter's mental health characteristics may be systematically biasing the rating of child emotional and behavioural problems. However, after conducting a variety of sensitivity analyses, we generally found little evidence that misclassification according to the maternal PRS to neuroticism was present. In fact, conflicting research suggests that the magnitude of these biases is often negligible (Olino et al., 2021). Some of this work indicates that differences in behaviours linked to reporters may be the result of mechanisms other than misclassification (e.g., cross situational differences in child behaviour [Achenbach et al., 1987], differential abilities of parents and teachers in recognising psychological problems, or direct and indirect genetic confounding [Warrington et al., 2021]) (Appendix S10 and Figure S7). Nevertheless, exploring evocative genetic effects is beyond the scope of this study.

Our study also has noticeable potential limitations. First, even though the findings obtained in the imputed datasets were comparable to those obtained using a complete case analysis, the validity of imputation relies on the assumption that data were missing‐at‐random. Another potential limitation of this study regards its generalisability. Whereas replicating this study in another longitudinal cohort with similar demographics may provide more robustness to the generalisability of these findings to WEIRD (Western, Educated, Industrialised, Rich, and Democratic) (Henrich et al., 2010) populations, the relevance of findings from this study to the full range of human variation and diversity is unknown (Gurven et al., 2013). Furthermore, the examination of internalising and externalising trajectories across childhood using observed scores assumes that the scale employed is measuring the same construct at all timepoints. A recent study (Speyer, Auyeung, et al., 2022) which systematically examined measurement invariance of the SDQ in the ALSPAC population from 4 to 16 years of age found that scalar invariance was achieved from age 7 onwards but was poor at age 4. However, to compare means over time based on observed scores (as opposed on latent factor means) requires ‘residual variance’ where residual variances are also equal over time. This was explicitly tested as part of our mixed‐effect model assumptions' check. However, a cautious interpretation of the results in toddlerhood is warranted as the changes observed at this age may be more reflective of differences in the measurement of the ‘behaviours’ than in the behaviours themselves. Lastly, although increases in the sample size of GWASs have led to increased statistical power, the overall variance explained by SNPs used to develop PRSs for neuroticism remains small (around 3%) (Luciano et al., 2018), limiting the ability of such PRSs to identify small to modest effects of genetic liability to neuroticism on subsequent emotional and behavioural outcomes. Additionally, effect sizes of the association between PRS and trajectories of internalising and externalising problems were small but aligned with previously presented effect sizes (Kwong et al., 2021).

Thus, our findings add to the current evidence (Speyer, Neaves, et al., 2022) that genetics may play a somewhat limited role in the onset and progression of internalising and externalising problems throughout childhood and adolescence. However, these small effect sizes highlight that the inclusion of environmental factors may improve the understanding of pathways to such emotional and behavioural difficulties. Environmental and social factors such as parental characteristics (e.g., depressive symptoms and parenting styles) and family income may explain a larger proportion of the variance of emotional and behavioural trajectories and/or moderate the effect of a child NEU PRS on subsequent childhood problems (Goodman, 2020; Goodman, Rouse, et al., 2011).

## CONCLUSION

Our comprehensive analyses using a large, well‐characterised birth cohort study of up to 6271 children suggest that a child NEU PRS associated with several psychological outcomes, showing some specificity for anxiety‐related problems. The NEU PRS also associated with the overall levels and rate of change of trajectories of internalising and externalising problems throughout childhood up to early adolescence. Future studies with complete parental genotype may help to further elucidate the causal nature of the associations reported. Finally, the use of trio designs combined with phenotypic measures of early‐life environmental factors may help to further disentangle the direct and indirect pathways leading from parental genotype to negative psychological outcomes in childhood.

## AUTHOR CONTRIBUTIONS

**Ilaria Costantini**: Conceptualization; Data curation; Formal analysis; Methodology; Software; Visualization; Writing – original draft; Writing – review & editing. **Hannah Sallis**: Data curation; Methodology; Writing – review & editing. **Kate Tilling**: Methodology; Supervision; Writing – review & editing. **Daniel Major‐Smith**: Methodology; Writing – review & editing. **Rebecca Pearson**: Funding acquisition; Methodology; Supervision; Writing – review & editing. **Daphne Kounali**: Methodology; Software; Supervision; Writing – review & editing.

## CONFLICT OF INTEREST STATEMENT

The authors declare that the research was conducted in the absence of any commercial or financial relationships that could be construed as a potential conflict of interest.

## ETHICAL CONSIDERATIONS

The studies involving human participants were reviewed and approved by Ethical approval for the study was obtained from the ALSPAC Law and Ethics Committee and Southwest National Health Service (NHS) Research Ethics Committee; participants gave written informed data consent. Written informed consent to participate in this study was provided by the participants' legal guardian/next of kin.

## Supporting information

Supporting Information S1

## Data Availability

The data analysed in this study is subject to the following licenses/restrictions: ALSPAC datasets can be accessed pending approval of research proposals. Requests to access these datasets should be directed to alspac-data@bristol.ac.uk, bbl-info@bristol.ac.uk.
