# Supplementary material for: Childhood trajectories of internalising and externalising problems associated with a polygenic risk score for neuroticism in a UK birth cohort study
Source: JCPP Adv. 2023 Feb 22;3(1):e12141. doi: 10.1002/jcv2.12141 (PMC10241477; doi:10.1002/jcv2.12141)
Supplement: Supplementary file 1 — Supporting Information S1 [file JCV2-3-e12141-s001.docx]

Supporting Information

# Childhood trajectories of internalising and externalising problems associated with a polygenic risk score for neuroticism in a UK birth cohort study

Ilaria Costantini, Hannah Sallis, Kate Tilling, Daniel Major-Smith, Rebecca M. Pearson, Daphne-Zacharenia Kounali

Contents

[Childhood trajectories of internalising and externalising problems associated with a polygenic risk score for neuroticism in a UK birth cohort study 1](#_Toc123215413)

[Appendix S1: Methods: Supplementary information on genotyping 5](#_Toc123215414)

[Genotype Information 5](#_Toc123215415)

[Appendix S2: Methods: Supplementary information on outcome measures 7](#_Toc123215416)

[Carey Infant Temperament Scales (CTSs) – 6 and 24 months 7](#_Toc123215417)

[Strengths and Difficulties Questionnaire (SDQ) – 4 to 11 years 7](#_Toc123215418)

[Axis-I Disorders – 7 and 11 years 7](#_Toc123215419)

[Locus of control – 8 years 8](#_Toc123215420)

[Self-Esteem – 8 years 8](#_Toc123215421)

[Intelligence Quotient (IQ) – 8 years 8](#_Toc123215422)

[Big Five – 13 years 8](#_Toc123215423)

[Appendix S3: Methods: Sensitivity analyses 10](#_Toc123215424)

[Exploring differential misclassification depending on maternal PRS 10](#_Toc123215425)

[Maternal PRS as confounding and potential pleiotropy of NEU PRS 11](#_Toc123215426)

[Appendix S4: Results 13](#_Toc123215427)

[Correlations between subscales of SDQ and between mother and teacher reports 26](#_Toc123215428)

[Cross-sectional analyses of the association between child NEU PRS and various psychological outcomes 28](#_Toc123215429)

[Positive and negative control analyses 28](#_Toc123215430)

[Appendix S5: Supplementary information on model selection and the selected linear mixed effect model 37](#_Toc123215431)

[Fitted linear mixed models 37](#_Toc123215432)

[Further information on the selected model 39](#_Toc123215433)

[Random Effects findings 39](#_Toc123215434)

[Teacher reporting 39](#_Toc123215435)

[Appendix S6: Results: Sensitivity analyses to explore potential differential misclassification of the outcome. 48](#_Toc123215436)

[Appendix S7: Results: Sensitivity analyses: exploring the causal nature of primary analyses 53](#_Toc123215437)

[Sensitivity analyses investigating the potential causal nature of the association between child NEU PRS and later psychological difficulties 53](#_Toc123215438)

[Appendix S8: Sensitivity analyses: Addressing missing data 53](#_Toc123215439)

[Sensitivity analyses investigating the potential bias arising from attrition 54](#_Toc123215440)

[Appendix S9: Sensitivity analyses: Using genome-wide significant p-value threshold NEU PRS 73](#_Toc123215441)

[Sensitivity analyses investigating the specificity of the association between child NEU PRS and the psychological outcomes via neuroticism and not via other pathways (i.e., minimising pleiotropy). 73](#_Toc123215442)

[TableS1. Number of SNPs included in the analyses at different P-value thresholds. 6](#_Toc123215443)

[TableS2. Descriptives of outcomes measures. 13](#_Toc123215444)

[TableS3. Descriptives of Development and Well-Being Assessment (DAWBA). 21](#_Toc123215445)

[TableS4. Distribution of socioeconomic, maternal, paternal, familial indicators and child psychosocial indicators in the original ALSPAC cohort in various sub-samples used in the study. 25](#_Toc123215446)

[TableS5. Correlations between SDQ scores across ages and reporters. 26](#_Toc123215447)

[TableS6. Separate linear regressions of child PRS toward neuroticism on various emotional and behavioural outcomes. Estimated with robust standard errors adjusted for sex, age and the first 5 principal components of genetic ancestry. 28](#_Toc123215448)

[TableS7. Separate ordinal regressions of a child PRS for neuroticism on various emotional and behavioural outcomes. 34](#_Toc123215449)

[TableS8. Model selection criteria SDQ models 37](#_Toc123215450)

[TableS9. Mean differences in low vs high child PRS at youngest compared to oldest child age by sex of the child and reporter. 44](#_Toc123215451)

[TableS10. Associations between maternal neuroticism PRS and child’s emotional and behavioural difficulties as rated by the teacher adjusted for the child’s neuroticism PRS. 49](#_Toc123215452)

[TableS11. Descriptives of “difference in score” variables. 50](#_Toc123215453)

[TableS12. Associations between maternal neuroticism PRS and “difference in score” outcomes (teacher - mother reported scores) unadjusted and adjusted for child neuroticism PRS. 51](#_Toc123215454)

[TableS13. OR for missing data in the main outcomes’ models. 55](#_Toc123215455)

[TableS14. Associations between child PRS for neuroticism and various psychological outcomes in multiple imputed datasets. 55](#_Toc123215456)

[TableS15. Associations between neuroticism PRS and all psychological outcomes, unadjusted and adjusted for maternal neuroticism PRS. 59](#_Toc123215457)

[TableS16. Associations between child PRS for neuroticism (threshold P < 5 × 10-8) and all psychological outcomes unadjusted and adjusted for maternal PRS for neuroticism. 74](#_Toc123215458)

[FigS1. Outcome measures time-points and reporter 8](#_Toc123215459)

[FigS2. Graphical representation of potential dependent differential misclassification. 10](#_Toc123215460)

[FigS3. Flow diagram of ALSPAC participants with genotype information and main outcome. 23](https://uob-my.sharepoint.com/personal/ic18563_bristol_ac_uk/Documents/temp/bristol/PhD_new/PRS%20and%20neuroticism/Supplementary%20material/AppendixJCPPA_APA.docx#_Toc123215461)

[FigS4. Normal Probability Plot. 40](#_Toc123215462)

[FigS5. Quantile-quantile (Q-Q) plot to assess normality of the residuals. 41](#_Toc123215463)

[FigS6. Trajectories of internalising and externalising problems by PRS levels across age when SDQ is teacher reported by sex of the child. 42](#_Toc123215464)

[FigS7. Directed Acyclic Graph (DAG) representing potential pathways through which children behave/experience emotional symptoms differently in presence of a parent. 86](#_Toc123215465)

# Appendix S1. Methods: Supplementary information on genotyping

## Genotype Information

ALSPAC children were genotyped using the Illumina HumanHap550 quad chip genotyping platforms. The resulting raw genome-wide data were subjected to standard quality control methods. Individuals were excluded on the basis of gender mismatches; minimal or excessive heterozygosity; disproportionate levels of individual missingness (>3%) and insufficient sample replication (IBD < 0.8). Population stratification was assessed by multidimensional scaling analysis and compared with Hapmap II (release 22) European descent (CEU), Han Chinese, Japanese and Yoruba reference populations; all individuals with non-European ancestry were removed. SNPs with a minor allele frequency of < 1%, a call rate of < 95%, or evidence for violations of Hardy-Weinberg equilibrium (P < 5 x 10^-7^) were removed. Cryptic relatedness was measured as proportion of identity by descent (IBD > 0.1). Related subjects that passed all other quality control thresholds were retained during subsequent phasing and imputation. 9,115 subjects and 500,527 SNPs passed these quality control filters.

ALSPAC mothers were genotyped using the Illumina human660W-quad array at Centre National de Génotypage (CNG) and genotypes were called with Illumina GenomeStudio. PLINK (v1.07) was used to carry out quality control measures on an initial set of 10,015 subjects and 557,124 directly genotyped SNPs. SNPs were removed if they displayed more than 5% missingness or a Hardy-Weinberg equilibrium P value of less than 1.0 x 10^-6^. Additionally, SNPs with a minor allele frequency of less than 1% were removed. Samples were excluded if they displayed more than 5% missingness, had indeterminate X chromosome heterozygosity, or extreme autosomal heterozygosity. Samples showing evidence of population stratification were identified by multidimensional scaling of genome-wide identity by state pairwise distances using the four HapMap populations as a reference, and then excluded. Cryptic relatedness was assessed using a IBD estimate of more than 0.125 which is expected to correspond to roughly 12.5% alleles shared IBD or a relatedness at the first cousin level. Related subjects that passed all other quality control thresholds were retained during subsequent phasing and imputation. 9,048 subjects and 526,688 SNPs passed these quality control filters.

After combining genotype data in the mothers and the children, SNPs with genotype missingness above 1% were removed due to poor quality (11,396 SNPs removed) and a further 321 subjects were removed due to potential ID mismatches. This resulted in a dataset of 17,842 subjects. Imputation of the target data was performed using Impute V2.2.2 against the 1000 genomes reference panel (Phase 1, Version 3) (all polymorphic SNPs excluding singletons), using all 2186 reference haplotypes (including non-Europeans). This gave 8,237 eligible children and 8,196 eligible mothers with available genotype data after exclusion of related subjects using cryptic relatedness measures described previously.

TableS1. Number of SNPs included in the analyses at different P-value thresholds.

| **P-value threshold** | **Child PRS** | **Maternal PRS** |
| --- | --- | --- |
| **5.00 x 10^-8^** | **113** | **114** |
| 1.00 x 10^-7^ | 140 | 141 |
| 1.00 x 10^-6^ | 235 | 236 |
| 1.00 x 10^-5^ | 492 | 493 |
| 0.0001 | 1,226 | 1,225 |
| 0.001 | 3,771 | 3,767 |
| 0.01 | 14,439 | 14,424 |
| **0.05** | **39,401** | **39,352** |
| 0.1 | 60,857 | 60,797 |
| 0.2 | 93,822 | 93,839 |
| 0.3 | 119,770 | 119,755 |
| 0.4 | 141,472 | 141,409 |
| 0.5 | 159,446 | 159,269 |
| 1 | 211,672 | 211,648 |

**Legend:** We have highlighted in bold the two P-value thresholds employed in this work.

# Appendix S2. Methods: Supplementary information on outcome measures

### Carey Infant Temperament Scales (CTSs) – *6 and 24 months*

The analyses were performed separately across every subscale relating to the temperament domains previously identified (Thomas & Chess, 1977): Activity (i.e., motor component in a child’s functioning), Rhythmicity (i.e., regularity; predictability in daily functions), Approach (i.e., initial response to a novel stimulus), Adaptability (i.e., behavioural flexibility in changing environments), Intensity (i.e., energy level of an emotional response), Mood (i.e., tone of overall affect: positive or negative), Persistence (i.e., continuation of activity in face of obstacles), Distractibility (i.e., effectiveness of extraneous stimuli in altering the direction of ongoing attention), and Threshold (i.e., the intensity level of stimulation that is necessary to evoke a discernible response) (Chess & Thomas, 2013). The measure was predominantly mother reported (94%) at 6 and 24 months of age. In the current sample, internal reliability as measured with Cronbach alpha was mixed, ranging from poor to good (0.36 to 0.73) when measured at 6 months and was more satisfactory when measured at 24 months (0.59 to 0.87).

### Strengths and Difficulties Questionnaire (SDQ) – *4 to 11 years*

The total score used here is a weighted sum of the components of the Hyperactivity, Emotional Symptoms, Conduct Problems, and Peer Problems scores. This questionnaire was used to assess child difficulties at 4 years (parent report – 98% mother report), 6 years (parent report – 97% mother report), 8 years (both parent – 97.8% mother report – and teacher report), 9 years (parent report – 97.6% mother report -), and at 11 years of age (both parent – 97.6% mother report – and teacher report). Internalising and externalising subscales were derived summing up the scores from the Emotional Symptoms and Peer Problems and the Hyperactivity and Conduct Problems subscales, respectively. Subscale scores were pro-rated if 1 or 2 items were missing ([www.sdqinfo.com/b4.html](http://www.sdqinfo.com/b4.html)). In the current sample, Cronbach’s alpha values for the total SDQ score were 0.80, 0.79, 0.82, 0.81, and 0.81, at age 4, 6, 8, 9, and 11 years, respectively when reported by the parent, and 0.87 and 0.88 at age 8 and 11 years, respectively, when reported by the teacher.

### Axis-I Disorders – *7 and 11 years*

The DAWBA has incorporated information from both parent and teacher reports for the ADHD and oppositional/conduct disorders, which should have minimised the possibility of differential misclassification. The advantages of using the ordered categorical variables over a binary classification of the scale are described elsewhere (Goodman et al., 2011).

### Locus of control – *8 years*

Children’s locus of control was assessed using an adaptation of the Children’s Nowicki Strickland Internal External scale (CNSIE) (Nowicki & Strickland, 1973). This form was administered to ALSPAC children when they were tested at 8 years of age. Items were read out loud by the examiners and the child was asked to respond yes or no. They were reminded of the confidentiality of the test and that there were no correct answers. Higher scores indicate a more external locus of control. In the current sample, the internal reliability as measured with Cronbach alpha was low (0.47).

### Self-Esteem *– 8 years*

Children’s self-worth and scholastic competence were assessed with a 12-item shortened form of Harter’s Self Perception Profile for Children (SPPC) (Harter, 1985) at around 8 years of age. In the current sample, internal reliability as measured with Cronbach alpha was satisfactory with values of 0.75, 0.69, and 0.65 for the total score, the scholastic self-esteem score, and the global self-esteem score, respectively.

### Intelligence Quotient (IQ) *– 8 years*

The Wechsler Intelligence Scale for Children (WISC-III UK) (Wechsler et al., 1992) was used to assess child’s IQ at 8 years of age.

**Variables used for positive and negative control analyses**

### Big Five – *13 years*

Here, we conducted a positive outcome control analysis to test the validity of the assumption that a NEU PRS was indeed associated with measured neuroticism (i.e., the emotional stability subscale of the Big Five). The Big Five questionnaire was measured as part of the TF2 clinic visit when the child was 13 years old. Higher scores on the emotional stability variable indicate higher emotional stability (i.e., lower neuroticism).

**Wear glasses -** ***8 and 11 years***

We conducted a negative control analysis to further test the validity of our exposure measure (i.e., NEU PRS) by checking that it was not associated with a variable we would not expect any association with: whether the child wore glasses or not. We used two timepoints: at around 8 and 11 years of age.

FigS1. Outcome measures time-points and reporter


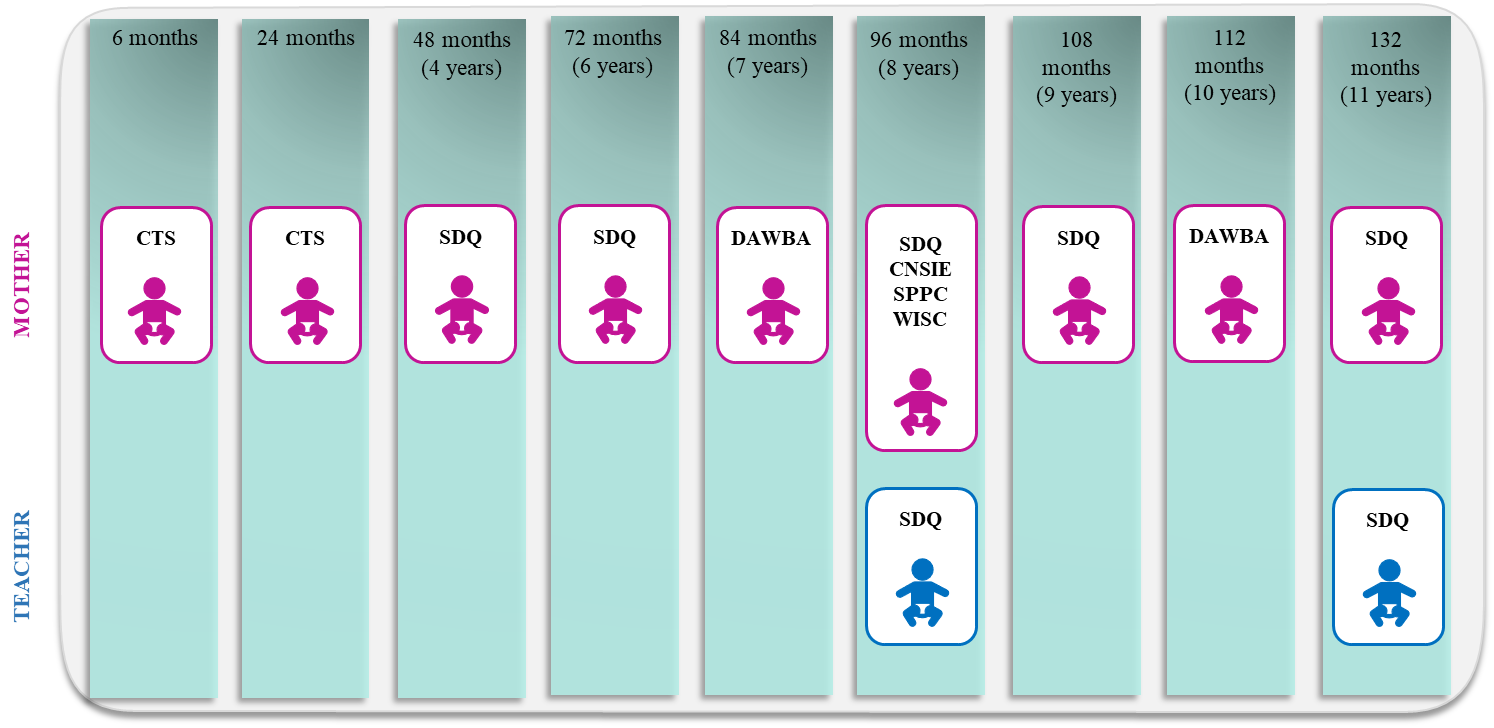


***Legend:*** *All outcomes are child measures, thus the child symbol. CTS: Carey Infant Temperament Scales (temperamental difficulties); SDQ: Strengths and Difficulties Questionnaire (emotional and behavioural problems); DAWBA: Development and Well-being Assessment (clinical disorders); CNSIE: Children’s Nowicki Strickland Internal External scale (locus of control); SPPC: Self Perception Profile for Children (self-esteem); WISC: Wechsler Intelligence Scale for Children (intelligence quotient). SDQ was reported twice by both main caregiver and teacher at comparable ages.*

# Appendix S3. Methods: Sensitivity analyses

### Exploring differential misclassification depending on maternal PRS

First, we used different methods to explore the potential presence of differential and dependent measurement error due to the maternal PRS.

Dependent differential misclassification is a systematic bias in the measurement of the outcome depending on a third factor. We illustrate potential measurement error or misclassification using signed directed acyclic graph (DAG) as suggested by VanderWeele et al (2012) (**FigS2**). Here, we hypothesised that mothers with a higher PRS for neuroticism may have been more sensitive to negative emotions in the child compared to women with a lower liability to neuroticism, leading to a higher score on scales measuring emotional and behavioural problems of the child. We employed different methods to explore the presence of differential misclassification of the child outcome (i.e., SDQ) dependent on a maternal PRS for neuroticism. We first used maternal genotype (N=7,826) as the exposure when assessing child’s behaviour as reported by the child’s teacher as an outcome while adjusting for child genotype. No evidence of association between the maternal PRS for neuroticism and the teacher rating of child behaviours, once adjusting for the child PRS for neuroticism, would suggest that the teacher rating is independent of the maternal PRS. Second, because of the availability of both maternal and teacher scoring of the SDQ at two different timepoints at approximately the same age (around 8 and 11 years of age), we explored the potential effect of differential misclassification dependent on the maternal PRS for neuroticism by generating a variable which represented the difference in score between the teacher and mother reported scores of child behaviours. This variable was obtained by subtracting the maternally reported score of child’s problems (measured with the SDQ at 8 and 11 years of age) from the teacher reported score of child’s problems (measured with the SDQ at 8 and 11 years of age). If this score had a mean value of 0, it suggested that no difference was present in the rating performed by the mother as compared to the teacher.

However, simply observing a difference in score does not imply that differential misclassification occurred. In fact, the difference could be representative of a real difference in the child behaviour (e.g., the child is clingier and less hyperactive at home than he or she is at school or vice-versa), resulting in a differential assessment by the parent and the teacher. However, if the difference in score was uniquely induced by a true difference in behaviour, we would expect it to be independent of the maternal PRS once adjusting for the child’s own PRS. Thus, we employed linear regression models adjusting for the child PRS to explore the association between the maternal PRS and the difference in score.

FigS2. Graphical representation of potential dependent differential misclassification.


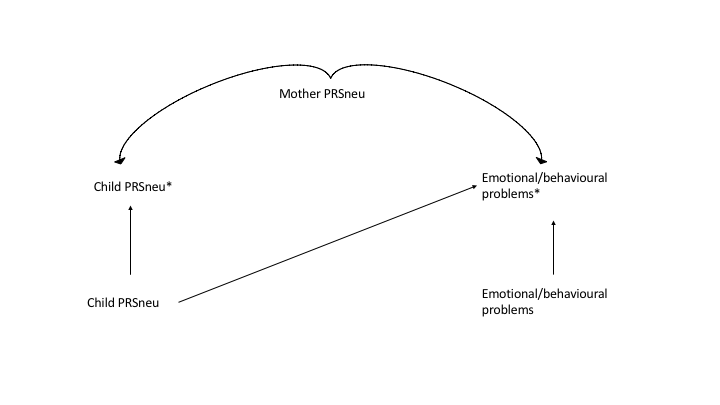


**Legend:** The variables with the asterisk* represent the measured exposure and the measured outcome. Maternal PRS for neuroticism is a variable leading to correlated/dependent measurement errors: lower versus higher levels of maternal PRS for neuroticism could differentially impact measurement of the child’s emotional and behavioural problems.

### Maternal PRS as confounding and potential pleiotropy of NEU PRS

We adjusted for maternal PRS in the separate multivariable linear regressions in order to account for a possible confounding effect of maternal NEU PRS on child’s outcomes. Then, we re-ran the cross-sectional models using a genome-wide significance threshold (P<5 x 10^-8^) for PRS construction. This could help to disentangle whether the observed effects of the neuroticism PRS are indeed acting through a liability to neuroticism as opposed to alternate pathways.

**Potential bias arising from attrition**

We investigated the potential for bias in our analyses due to levels of attrition in the outcomes using 100 generated imputed datasets estimated via multiple imputation through chained equations (MICE) (Royston, 2005, 2007; Sterne et al., 2009).

In this study, sample attrition on the outcome of interest was moderately high, ranging from 20% to 55%; in addition, after analysing proportions of missingness according to different socio-demographic indicators and exploring variables associated with missingness, we found evidence that the probability of having missing data depended on observed values (Missing At Random – MAR) (**TableS4**).

Therefore, we employed multiple imputation through chained equations (MICE), also known as fully conditional specification, on 100 imputed datasets with 20 cycles of regression switching for two separate cross-sectional analysis models (i.e., one model with temperament - CTSs - and one model with emotional and behavioural difficulties - SDQs - as outcomes) using the ice command in Stata 16(Royston, 2005, 2007; Sterne et al., 2009). Samples were restricted to those children who had genetic data and at least one outcome variable of the repeated measures available. We used the “*by*” command to perform imputations separately on boys and girls. When imputing the outcomes, we included all the variables of our models (including covariates and past and future measures of the outcome) and auxiliary variables which were associated with missingness (**TableS15**). Imputations on the DAWBA outcome were not performed because of the low number of cases.

MICE was employed under the assumption that data were MAR. Including auxiliary variables is recommended to make the MAR assumption more plausible (Hughes et al., 2019), however we kept the number of covariates included relatively small, selecting those with little/no missing values and that were strongly associated with levels of missingness in our models (Lee et al., 2020).

As we were not able to employ MICE on all the analyses (e.g., DAWBA and sensitivity analysis using the maternal PRS for neuroticism) that we performed in the complete case analysis (CCA), the imputed results are presented only as a sensitivity analysis. We examined consistency across results to explore whether our complete case findings were biased because of missing data (Hughes et al., 2019).

# Appendix S4. Results

TableS2. Descriptives of outcomes measures.

| **Variable** | **Mean** | **SD** | **Min** | **Max** | **Mean (SD) age at completion in months** | **Cronbach’s alphas** | **Reporter** | **All Participants with Measure** | **Genotyped Participants** |
| --- | --- | --- | --- | --- | --- | --- | --- | --- | --- |
| Sex |  |  | 0 | 1 |  |  |  | 15,039 (48.86% females) | 7,847 (48.74% females) |
| Carey Activity | 40.13 | 6.34 | 13 | 60 | 6.09 (0.31) | 0.56 | Mother | 10,503 | 6,275 |
| Carey Rhythm | 15.97 | 6.93 | 0 | 46 | 6.09 (0.31) | 0.73 | Mother | 10,500 | 6,273 |
| Carey Approach | 15.01 | 6.36 | 0 | 43 | 6.09 (0.31) | 0.71 | Mother | 10,495 | 6,270 |
| Carey Adapt | 14.00 | 5.71 | 0 | 42 | 6.09 (0.31) | 0.59 | Mother | 10,508 | 6,275 |
| Carey Intensity | 25.03 | 5.59 | 4 | 49 | 6.09 (0.31) | 0.36 | Mother | 10,494 | 6,273 |
| Carey Mood | 15.81 | 5.91 | 0 | 44 | 6.09 (0.31) | 0.61 | Mother | 10,496 | 6,271 |
| Carey Persist | 13.57 | 5.11 | 0 | 34 | 6.09 (0.31) | 0.60 | Mother | 10,504 | 6,273 |
| Carey Distract | 14.02 | 5.61 | 0 | 36 | 6.09 (0.31) | 0.58 | Mother | 10,506 | 6,275 |
| Carey Threshold | 27.51 | 6.06 | 3 | 50 | 6.09 (0.31) | 0.49 | Mother | 10,490 | 6,268 |
| Carey Activity | 23.15 | 4.54 | 3 | 36 | 24.44 (1.13) | 0.59 | Mother | 10,327 | 6,264 |
| Carey Rhythm | 12.79 | 5.76 | 0 | 40 | 24.44 (1.13) | 0.80 | Mother | 10,321 | 6,261 |
| Carey Approach | 20.23 | 7.68 | 0 | 44 | 24.44 (1.13) | 0.83 | Mother | 10,318 | 6,257 |
| Carey Adapt | 12.79 | 4.18 | 0 | 28 | 24.44 (1.13) | 0.75 | Mother | 10,272 | 6,237 |
| Carey Intensity | 21.40 | 4.54 | 4 | 36 | 24.44 (1.13) | 0.81 | Mother | 10,317 | 6,259 |
| Carey Mood | 18.08 | 5.69 | 1 | 48 | 24.44 (1.13) | 0.83 | Mother | 10,325 | 6,263 |
| Carey Persist | 16.26 | 4.89 | 0 | 35 | 24.44 (1.13) | 0.82 | Mother | 10,312 | 6,256 |
| Carey Distract | 24.54 | 4.68 | 0 | 40 | 24.44 (1.13) | 0.87 | Mother | 10,319 | 6,259 |
| Carey Threshold | 19.05 | 4.40 | 0 | 32 | 24.44 (1.13) | 0.72 | Mother | 10,327 | 6,264 |
| IQ (WISC) | 103.97 | 16.54 | 45 | 151 | 103.83 (3.92) | - | Child | 7,354 | 5,297 |
| Locus of control | 5.99 | 2.08 | 0 | 12 | 103.83 (3.92) | 0.47 | Child | 6,380 | 4,601 |
| **Variable** | **Median** | **IQR** | **Min** | **Max** | **Mean (SD) age at completion** | **Alpha** | **Reporter** | **All Participants with Measure** | **Genotyped Participants** |
| SDQ total score | 8 | 6 - 12 | 0 | 33 | 48 (1.44) | 0.80 | Mother | 9,457 | 5,911 |
| SDQ total score | 7 | 4 - 10 | 0 | 33 | 81.45 (1.36) | 0.79 | Mother | 8,404 | 5,514 |
| SDQ total score | 7 | 4 - 11 | 0 | 38 | 98.43 (3.09) | 0.82 | Mother | 7,785 | 5,287 |
| SDQ total score | 4 | 2 - 9 | 0 | 37 | 99.92 (3.74) | 0.87 | Teacher | 6,368 | 3,589 |
| SDQ total score | 6 | 3 - 9 | 0 | 35 | 115.82 (1.57) | 0.81 | Mother | 8,040 | 5,520 |
| SDQ total score | 4 | 1 - 9 | 0 | 37 | 133.99 (3.89) | 0.88 | Teacher | 7,666 | 4,273 |
| SDQ total score | 5 | 3 - 9 | 0 | 34 | 140.62 (1.64) | 0.81 | Mother | 7,354 | 5,131 |
| SDQ emotional subscale | 1 | 0 - 2 | 0 | 10 | 48 (1.44) | 0.68 | Mother | 9,500 | 5,932 |
| SDQ emotional subscale | 1 | 0 - 2 | 0 | 10 | 81.45 (1.36) | 0.63 | Mother | 8,423 | 5,521 |
| SDQ emotional subscale | 1 | 0 - 3 | 0 | 10 | 98.43 (3.09) | 0.69 | Mother | 7,795 | 5,286 |
| SDQ emotional subscale | 1 | 0 - 2 | 0 | 10 | 99.92 (3.74) | 0.80 | Teacher | 6,369 | 3,590 |
| SDQ emotional subscale | 1 | 0 - 2 | 0 | 10 | 115.82 (1.57) | 0.68 | Mother | 8,052 | 5,528 |
| SDQ emotional subscale | 0 | 0 - 2 | 0 | 10 | 133.99 (3.89) | 0.80 | Teacher | 7,665 | 4,273 |
| SDQ emotional subscale | 1 | 0 - 2 | 0 | 10 | 140.62 (1.64) | 0.67 | Mother | 7,340 | 5,121 |
| SDQ hyperactivity subscale | 4 | 2 - 5 | 0 | 10 | 48 (1.44) | 0.76 | Mother | 9,492 | 5,931 |
| SDQ hyperactivity subscale | 3 | 1 - 5 | 0 | 10 | 81.45 (1.36) | 0.77 | Mother | 8,409 | 5,522 |
| SDQ hyperactivity subscale | 3 | 1 - 5 | 0 | 10 | 98.43 (3.09) | 0.80 | Mother | 7,796 | 5,286 |
| SDQ hyperactivity subscale | 2 | 0 - 4 | 0 | 10 | 99.92 (3.74) | 0.87 | Teacher | 6,369 | 3,590 |
| SDQ hyperactivity subscale | 3 | 1 - 4 | 0 | 10 | 115.82 (1.57) | 0.76 | Mother | 8,069 | 5,542 |
| SDQ hyperactivity subscale | 1.5 | 0 - 4 | 0 | 10 | 133.99 (3.89) | 0.88 | Teacher | 7,666 | 4,273 |
| SDQ hyperactivity subscale | 2 | 1 - 4 | 0 | 10 | 140.62 (1.64) | 0.77 | Mother | 7,339 | 5,121 |
| SDQ conduct subscale | 2 | 1 - 3 | 0 | 10 | 48 (1.44) | 0.58 | Mother | 9,486 | 5,928 |
| SDQ conduct subscale | 1 | 0 - 2 | 0 | 10 | 81.45 (1.36) | 0.55 | Mother | 8,433 | 5,529 |
| SDQ conduct subscale | 1 | 0 - 2 | 0 | 10 | 98.43 (3.09) | 0.59 | Mother | 7,798 | 5,287 |
| SDQ conduct subscale | 0 | 0 - 1 | 0 | 10 | 99.92 (3.74) | 0.7 | Teacher | 6,365 | 3,588 |
| SDQ conduct subscale | 1 | 0 - 2 | 0 | 10 | 115.82 (1.57) | 0.57 | Mother | 8,069 | 5,541 |
| SDQ conduct subscale | 0 | 0 - 1 | 0 | 10 | 133.99 (3.89) | 0.77 | Teacher | 7,661 | 4,272 |
| SDQ conduct subscale | 1 | 0 - 2 | 0 | 10 | 140.62 (1.64) | 0.59 | Mother | 7,357 | 5,131 |
| SDQ prosocial subscale | 7 | 6 - 9 | 0 | 10 | 48 (1.44) | 1 | Mother | 9,487 | 5,928 |
| SDQ prosocial subscale | 9 | 7 - 10 | 0 | 10 | 81.45 (1.36) | 0.72 | Mother | 8,432 | 5,528 |
| SDQ prosocial subscale | 8 | 7 - 10 | 0 | 10 | 98.43 (3.09) | 1 | Mother | 7,804 | 5,291 |
| SDQ prosocial subscale | 8 | 6 - 10 | 0 | 10 | 99.92 (3.74) | 0.87 | Teacher | 6,364 | 3,586 |
| SDQ prosocial subscale | 9 | 7 - 10 | 0 | 10 | 115.82 (1.57) | 0.68 | Mother | 8,076 | 5,549 |
| SDQ prosocial subscale | 9 | 6 - 10 | 0 | 10 | 133.99 (3.89) | 0.87 | Teacher | 7,666 | 4,273 |
| SDQ prosocial subscale | 9 | 7 - 10 | 0 | 10 | 140.62 (1.64) | 0.69 | Mother | 7,366 | 5,135 |
| SDQ peer problems subscale | 1 | 0 - 2 | 0 | 9 | 48 (1.44) | 0.56 | Mother | 9,499 | 5,931 |
| SDQ peer problems subscale | 1 | 0 - 2 | 0 | 10 | 81.45 (1.36) | 0.59 | Mother | 8,428 | 5,525 |
| SDQ peer problems subscale | 1 | 0 - 2 | 0 | 10 | 98.43 (3.09) | 0.61 | Mother | 7,794 | 5,287 |
| SDQ peer problems subscale | 0 | 0 - 2 | 0 | 10 | 99.92 (3.74) | 0.76 | Teacher | 6,369 | 3,590 |
| SDQ peer problems subscale | 1 | 0 - 2 | 0 | 10 | 115.82 (1.57) | 0.63 | Mother | 8,059 | 5,536 |
| SDQ peer problems subscale | 0 | 0 - 2 | 0 | 10 | 133.99 (3.89) | 0.78 | Teacher | 7,666 | 4,273 |
| SDQ peer problems subscale | 1 | 0 - 2 | 0 | 10 | 140.62 (1.64) | 0.65 | Mother | 7,362 | 5,133 |
| SDQ Internalising subscale | 3 | 1 – 4 | 0 | 16 | 48 (1.44) | 0.7 | Mother | 9,495 | 5,929 |
| SDQ Internalising subscale | 2 | 1 – 4 | 0 | 17 | 81.45 (1.36) | 0.69 | Mother | 8,415 | 5,517 |
| SDQ Internalising subscale | 2 | 1 – 4 | 0 | 19 | 98.43 (3.09) | 0.72 | Mother | 7,789 | 5,285 |
| SDQ Internalising subscale | 1 | 0 – 4 | 0 | 19 | 99.92 (3.74) | 0.82 | Teacher | 6,369 | 3,590 |
| SDQ Internalising subscale | 2 | 1 – 4 | 0 | 19 | 115.82 (1.57) | 0.73 | Mother | 8,032 | 5,516 |
| SDQ Internalising subscale | 1 | 0 – 4 | 0 | 19 | 133.99 (3.89) | 0.84 | Teacher | 7,665 | 4,273 |
| SDQ Internalising subscale | 2 | 1 – 4 | 0 | 20 | 140.62 (1.64) | 0.73 | Mother | 7,333 | 5,116 |
| SDQ Externalising subscale | 6 | 4 – 8 | 0 | 19 | 48 (1.44) | 0.78 | Mother | 9,459 | 5,913 |
| SDQ Externalising subscale | 5 | 2 – 7 | 0 | 19 | 81.45 (1.36) | 0.77 | Mother | 8,392 | 5,507 |
| SDQ Externalising subscale | 4 | 2 – 7 | 0 | 20 | 98.43 (3.09) | 0.80 | Mother | 7,791 | 5,283 |
| SDQ Externalising subscale | 2 | 0 - 5 | 0 | 20 | 99.92 (3.74) | 0.87 | Teacher | 6,360 | 3,585 |
| SDQ Externalising subscale | 4 | 2 – 6 | 0 | 20 | 115.82 (1.57) | 0.77 | Mother | 8,032 | 5,519 |
| SDQ Externalising subscale | 2 | 0 - 5 | 0 | 20 | 133.99 (3.89) | 0.89 | Teacher | 7,661 | 4,272 |
| SDQ Externalising subscale | 3 | 2 – 6 | 0 | 20 | 140.62 (1.64) | 0.78 | Mother | 7,327 | 5,115 |
| Self-Esteem Scholastic | 17 | 15 – 20 | 6 | 24 | 103.83 (3.92) | 0.69 | Child | 6,952 | 5,018 |
| Self-Esteem Global | 20 | 17 – 22 | 6 | 24 | 103.83 (3.92) | 0.65 | Child | 6,941 | 5,011 |

**Legend:** *SDQ Prosocial Score* (this provides a score ranging from 0 to 10, with a higher score indicating better behaviour of the child), *SDQ Hyperactivity Score* (this provides a score ranging from 0 to 10, with a higher score indicating a more hyperactive behaviour of the child), *SDQ Emotional Symptoms Score:* (this provides a score ranging from 0 to 10, with a higher the score indicating more emotional behaviour of the child), *SDQ Conduct Problems Score* (this provides a score ranging from 0 to 10, with a higher score indicating worse behaviour of the child), and *SDQ Peer Problems Score* (this provides a score ranging from 0 to 10, with a higher score indicating that the child behaves in a more pro-social manner with other children). The *SDQ Internalising subscale* is the sum of the Emotional and Peer Problems scales (this provides a score ranging from 0 to 20, with a higher score indicating more internalising problems) and the *SDQ Externalising subscale* is the sum of the Hyperactivity and Conduct Problems scales (this provides a score ranging from 0 to 20, with a higher score indicating more externalising problems).

TableS3. Descriptives of Development and Well-Being Assessment (DAWBA).

| **Variable** | **Median** | | **IQR** | **Min** | | | **Max** | **Mean (SD) age at completion** | | **All Participants with Measure** | | **Genotyped Participants** |
| --- | --- | --- | --- | --- | --- | --- | --- | --- | --- | --- | --- | --- |
| Attention deficit hyperactivity disorder (ADHD) | 0 | 0 – 1 | | | 0 | 5 | | | 91.88 (1.70) | | 8,207 | 5,496 |
| Hyperkinesis | 0 | 0 – 1 | | | 0 | 5 | | | 91.88 (1.70) | | 8,207 | 5,496 |
| Conduct disorder | 1 | 1 – 2 | | | 1 | 5 | | | 91.88 (1.70) | | 8,115 | 5,437 |
| Depressive disorder | 0 | 0 – 1 | | | 0 | 4 | | | 91.88 (1.70) | | 8,089 | 5,427 |
| Generalised anxiety disorder (GAD) | 2 | 1 – 2 | | | 1 | 4 | | | 91.88 (1.70) | | 8,203 | 5,495 |
| Obsessive compulsive disorder (OCD) | 0 | 0 – 0 | | | 0 | 4 | | | 91.88 (1.70) | | 8,234 | 5,512 |
| Oppositional disorder | 2 | 1 – 2 | | | 1 | 5 | | | 91.88 (1.70) | | 8,183 | 5,481 |
| Separation anxiety | 1 | 1 – 1 | | | 1 | 5 | | | 91.88 (1.70) | | 8,215 | 5,502 |
| Separation anxiety (ICD-10) | 0 | 0 – 0 | | | 0 | 4 | | | 91.88 (1.70) | | 8,215 | 5,502 |
| Social phobia | 0 | 0 – 1 | | | 0 | 4 | | | 91.88 (1.70) | | 8,222 | 5,505 |
| Specific phobias | 1 | 0 – 1 | | | 0 | 4 | | | 91.88 (1.70) | | 8,236 | 5,509 |
| Any disorder | 2 | 2 – 2 | | | 1 | 5 | | | 91.88 (1.70) | | 8,252 | 5,519 |
| Any emotional disorder | 2 | 1 – 2 | | | 1 | 5 | | | 91.88 (1.70) | | 8,252 | 5,519 |
| Any anxiety disorder | 2 | 1 – 2 | | | 1 | 5 | | | 91.88 (1.70) | | 8,252 | 5,519 |
| Any behavioural disorder | 2 | 2 – 2 | | | 1 | 5 | | | 91.88 (1.70) | | 8,183 | 5,481 |
| Attention deficit hyperactivity disorder (ADHD) | 0 | 0 – 1 | | | 0 | 5 | | | 128.66 (1.78) | | 7,785 | 5,339 |
| Conduct disorder | 1 | 1 – 2 | | | 1 | 5 | | | 128.66 (1.78) | | 7,687 | 5,282 |
| Depressive disorder | 0 | 0 – 1 | | | 0 | 5 | | | 128.66 (1.78) | | 7,657 | 5,267 |
| Generalised anxiety disorder (GAD) | 2 | 1 – 2 | | | 1 | 4 | | | 128.66 (1.78) | | 7,773 | 5,334 |
| Obsessive compulsive disorder (OCD) | 0 | 0 – 0 | | | 0 | 4 | | | 128.66 (1.78) | | 7,801 | 5,352 |
| Oppositional disorder | 2 | 1 – 2 | | | 1 | 5 | | | 128.66 (1.78) | | 7,759 | 5,326 |
| Separation anxiety | 1 | 1 – 1 | | | 1 | 5 | | | 128.66 (1.78) | | 7,527 | 5,160 |
| Social phobia | 0 | 0 – 1 | | | 0 | 4 | | | 128.66 (1.78) | | 7,797 | 5,347 |
| Specific phobias | 1 | 0 – 1 | | | 0 | 4 | | | 128.66 (1.78) | | 7,788 | 5,338 |
| Any disorder | 2 | 1 – 2 | | | 1 | 5 | | | 128.66 (1.78) | | 7,824 | 5,362 |
| Any emotional disorder | 2 | 1 – 2 | | | 1 | 5 | | | 128.66 (1.78) | | 7,823 | 5,361 |
| Any anxiety disorder | 2 | 1 – 2 | | | 1 | 5 | | | 128.66 (1.78) | | 7,823 | 5,361 |
| Any behavioural disorder | 2 | 1 – 2 | | | 1 | 5 | | | 128.66 (1.78) | | 7,759 | 5,326 |

FigS3. Flow diagram of ALSPAC participants with genotype information and main outcome.

**
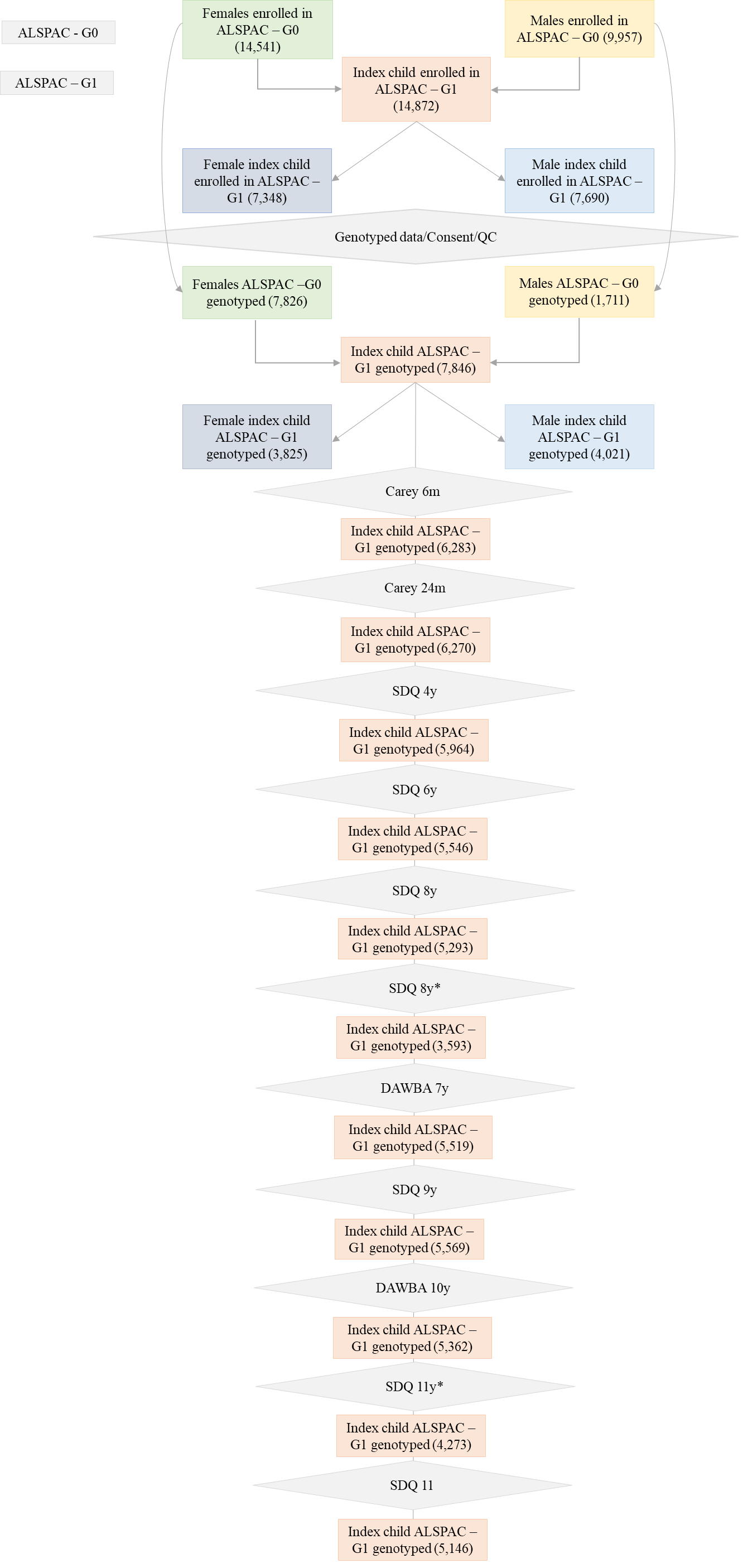
**

TableS4. Distribution of socioeconomic, maternal, paternal, familial indicators and child psychosocial indicators in the original ALSPAC cohort in various sub-samples used in the study.

| Variables | | Total ALSPAC-G1 sample (N=14,872) % | Sample with Carey at 6 months and covariates (N= 6,248) % | Sample with Carey at 24 months and covariates (N=6,225) % | Sample with SDQ at 4y and covariates (N= 5,788) % | Sample with SDQ at 6y and covariates (N= 5,435) % | Sample with SDQ at 8y and covariates (N= 5,276) % | Sample with SDQ at 8y* and covariates (N= 3,575) % | Sample with SDQ at 9y and covariates (N=5,488) % | Sample with SDQ at 11y* and covariates (N=4,269) % | Sample with SDQ at 11y and covariates (N= 5,091) % | | Sample with Dawba at 7y and covariates (N=5,260) % | | Sample with Dawba at 10y and covariates (N= 4,970) % | |
| --- | --- | --- | --- | --- | --- | --- | --- | --- | --- | --- | --- | --- | --- | --- | --- | --- |
| **Maternal education** | | | | | | | | | | | |  | |  | |  |
| A level or higher | 12.89 | 17.71 | 17.05 | 17.15 | 17.26 | 18.61 | 15.71 | 18.44 | 14.57 | 19.25 | | 18.42 | | 19.08 | |  |
| O level | 22.38 | 25.87 | 26.23 | 26.36 | 26.81 | 27.44 | 25.68 | 27.05 | 24.88 | 27.20 | | 26.93 | | 27.25 | |  |
| <O level | 64.72 | 57.41 | 56.73 | 56.49 | 55,26 | 53.95 | 58.60 | 54.51 | 60.55 | 53.55 | | 54.65 | | 53.67 | |  |
| N sample | 12,479 | 6,091 | 6,089 | 5,661 | 5,337 | 5,019 | 3,360 | 5,142 | 3,863 | 4,775 | | 5,129 | | 4,654 | |  |
| **Maternal Social Class** | | | | | | | | | | | | | | | | |
| High | 37.36 | 42.03 | 42.51 | 42.75 | 43.64 | 44.66 | 40.74 | 44.96 | 39.65 | 44.73 | | 43.84 | | 44.86 | |  |
| Low | 62.64 | 57,97 | 57.49 | 57.25 | 56.36 | 55.34 | 59.26 | 55.14 | 60.35 | 55.27 | | 56.16 | | 55.14 | |  |
| N sample | 10, 107 | 5,229 | 5,239 | 4,898 | 4,665 | 4,402 | 2,857 | 4,485 | 3,286 | 4,178 | | 4,489 | | 4,070 | |  |
| **Mother smoked in first 3 Months pregnancy** | | | | | | | | | | | | | | | | |
| No | 74.80 | 80.45 | 80.97 | 80.99 | 81.73 | 82.15 | 80.13 | 82.59 | 78.98 | 82.70 | | 81.92 | | 82.75 | |  |
| Yes | 25.20 | 19.55 | 19.03 | 19.01 | 18.27 | 18.85 | 19.87 | 17.41 | 21.02 | 17.30 | | 18.08 | | 17.25 | |  |
| N sample | 13,345 | 6,159 | 6,155 | 5,719 | 5,375 | 5,064 | 3,452 | 5,186 | 3,963 | 4,816 | | 5,177 | | 4,708 | |  |

**Legend:** variables with * refer to teacher reported measures.

## Correlations between subscales of SDQ and between mother and teacher reports

Pairwise Pearson correlations between the total score of SDQ across ages varied between 0.47 to 0.73 when reported by the mother and 0.57 when reported by the teacher. Inter-reporter correlations on total SDQ varied between 0.20 to 0.45. Correlation scores for internalising problems ranged from 0.32 to 0.63 when reported by the mother, 0.40 when reported by the teacher and between 0.13 to 0.33 when comparing inter-reporters. Correlation scores for externalising problems ranged from 0.48 to 0.74 when reported by the mother, 0.63 when reported by the teacher and ranged from 0.24 to 0.46 when comparing inter-reporters (**TableS5**). Cross-informant correlations were similar to those found by Achenbach et al. (1987) in a meta-analysis which explored correlations of emotional and behavioural problems of children and adolescents when using multiple reporters.

TableS5. Correlations between SDQ scores across ages and reporters.

| **Variables** | **SDQ_4y** | **SDQ_6y** | **SDQ_8y** | **SDQ_8y*** | **SDQ_9y** | **SDQ_11y*** | **SDQ_11y** |
| --- | --- | --- | --- | --- | --- | --- | --- |
| SDQ_4y | 1 |  |  |  |  |  |  |
| SDQ_6y | 0.58 | 1 |  |  |  |  |  |
| SDQ_8y | 0.57 | 0.73 | 1 |  |  |  |  |
| SDQ_8y* | 0.25 | 0.37 | 0.43 | 1 |  |  |  |
| SDQ_9y | 0.52 | 0.70 | 0.73 | 0.42 | 1 |  |  |
| SDQ_11y* | 0.20 | 0.31 | 0.35 | 0.57 | 0.37 | 1 |  |
| SDQ_11y | 0.47 | 0.64 | 0.67 | 0.42 | 0.72 | 0.43 | 1 |
| **Variables** | **Int_4y** | **Int_6y** | **Int_8y** | **Int_8y*** | **Int_9y** | **Int_11y*** | **Int_11y** |
| Int_4y | 1 |  |  |  |  |  |  |
| Int_6y | 0.46 | 1 |  |  |  |  |  |
| Int_8y | 0.44 | 0.63 | 1 |  |  |  |  |
| Int_8y* | 0.16 | 0.26 | 0.33 | 1 |  |  |  |
| Int_9y | 0.38 | 0.56 | 0.62 | 0.34 | 1 |  |  |
| Int_11y* | 0.13 | 0.22 | 0.27 | 0.40 | 0.31 | 1 |  |
| Int_11y | 0.32 | 0.51 | 0.54 | 0.33 | 0.61 | 0.35 | 1 |
| **Variables** | **Ext_4y** | **Ext_6y** | **Ext_8y** | **Ext_8y*** | **Ext_9y** | **Ext_11y*** | **Ext_11y** |
| Ext_4y | 1 |  |  |  |  |  |  |
| Ext_6y | 0.59 | 1 |  |  |  |  |  |
| Ext_8y | 0.58 | 0.73 | 1 |  |  |  |  |
| Ext_8y* | 0.28 | 0.42 | 0.46 | 1 |  |  |  |
| Ext_9y | 0.54 | 0.71 | 0.74 | 0.45 | 1 |  |  |
| Ext_11y* | 0.24 | 0.34 | 0.38 | 0.63 | 0.39 | 1 |  |
| Ext_11y | 0.48 | 0.65 | 0.69 | 0.45 | 0.73 | 0.45 | 1 |

**Legend:** SDQ_4y to SDQ_11y correspond to the total score on the SDQ; Int_4y to Int_11y correspond to the internalising scale of the SDQ (emotional subscale + peer problems subscale); Ext_4y to Ext_11y correspond to the externalising scale of the SDQ (hyperactivity subscale + conduct problems subscale).

Asterisk* indicates when the SDQ was reported by the teacher.

## Cross-sectional analyses of the association between child NEU PRS and various psychological outcomes

## Positive and negative control analyses

We found strong evidence of an inverse association between child NEU PRS and emotional stability (β_emotional stability_=-0.75, 95% CI: -0.96 to -0.55), supporting the internal validity of our exposure (i.e., positive control). Similarly, we found little association between our exposure and child wearing glasses at 8 and 10 years of age (OR_8y_=0.92, 95% CI_8y_=0.81 to 1.03 and OR_10y_=0.99, 95% CI_10y_=0.89 to 1.10) (i.e., negative control).

TableS6. Separate linear regressions of child PRS toward neuroticism on various emotional and behavioural outcomes. Estimated with robust standard errors adjusted for sex, age and the first 5 principal components of genetic ancestry.

| **Outcome variable** | **Age** | **Report** | **N** | **Beta** | **95% CIs** | **P-value** |
| --- | --- | --- | --- | --- | --- | --- |
| Prorated score of Carey – Activity subscale | 6 months | Mother | 6,271 | -0.16 | (-0.33, 0.00) | 0.057 |
| Prorated score of Carey – Rhythm subscale | 6 months | Mother | 6,269 | 0.08 | (-0.10, 0.26) | 0.393 |
| Prorated score of Carey – Approach subscale | 6 months | Mother | 6,266 | 0.18 | (0.01, 0.35) | 0.034 |
| Prorated score of Carey – Adaptability subscale | 6 months | Mother | 6,271 | 0.11 | (-0.04, 0.27) | 0.139 |
| Prorated score of Carey – Intensity subscale | 6 months | Mother | 6,269 | 0.01 | (-0.14, 0.16) | 0.896 |
| Prorated score of Carey – Mood subscale | 6 months | Mother | 6,267 | 0.03 | (-0.12, 0.19) | 0.665 |
| Prorated score of Carey – Persistence subscale | 6 months | Mother | 6,269 | -0.03 | (-0.16, 0.11) | 0.716 |
| Prorated score of Carey – Distract subscale | 6 months | Mother | 6,271 | 0.07 | (-0.07, 0.22) | 0.323 |
| Prorated score of Carey – Threshold | 6 months | Mother | 6,264 | 0.21 | (0.05, 0.37) | 0.010 |
| Prorated score of Carey – Activity subscale | 24 months | Mother | 6,260 | 0.08 | (-0.04, 0.20) | 0.174 |
| Prorated score of Carey – Rhythm subscale | 24 months | Mother | 6,257 | 0.25 | (0.09, 0.40) | 0.002 |
| Prorated score of Carey – Approach subscale | 24 months | Mother | 6,253 | 0.17 | (-0.03, 0.36) | 0.092 |
| Prorated score of Carey – Adaptability subscale | 24 months | Mother | 6,233 | 0.15 | (0.04, 0.27) | 0.006 |
| Prorated score of Carey – Intensity subscale | 24 months | Mother | 6,255 | 0.14 | (0.02, 0.26) | 0.027 |
| Prorated score of Carey – Mood subscale | 24 months | Mother | 6,259 | 0.21 | (0.06, 0.36) | 0.005 |
| Prorated score of Carey – Persistence subscale | 24 months | Mother | 6,252 | 0.20 | (0.07, 0.32) | 0.002 |
| Prorated score of Carey – Distract subscale | 24 months | Mother | 6,255 | 0.07 | (-0.05, 0.20) | 0.260 |
| Prorated score of Carey – Threshold | 24 months | Mother | 6,260 | -0.10 | (-0.21, 0.02) | 0.113 |
| Prosocial prorated subscale of SDQ | 4 years | Mother | 5,803 | -0.04 | (-0.10, 0.01) | 0.117 |
| Hyperactive prorated subscale of SDQ | 4 years | Mother | 5,808 | 0.06 | (-0.01, 0.12) | 0.074 |
| Emotional prorated subscale of SDQ | 4 years | Mother | 5,807 | 0.05 | (0.01, 0.09) | 0.014 |
| Conduct prorated subscale of SDQ | 4 years | Mother | 5,803 | 0.08 | (0.04, 0.11) | 7.46 x 10^-6^ |
| Peer problems prorated subscale of SDQ | 4 years | Mother | 5,806 | 0.09 | (0.05, 0.13) | 1.03 x 10^-5^ |
| Total prorated score of SDQ | 4 years | Mother | 5,788 | 0.27 | (0.15, 0.40) | 2.30 x 10^-5^ |
| Prosocial prorated subscale of SDQ | 6 years | Parent | 5,524 | -0.01 | (-0.06, 0.04) | 0.59 |
| Hyperactive prorated subscale of SDQ | 6 years | Parent | 5,518 | 0.08 | (0.02, 0.15) | 0.011 |
| Emotional prorated subscale of SDQ | 6 years | Parent | 5,517 | 0.12 | (0.08, 0.17) | 1.73 x 10^-7^ |
| Conduct prorated subscale of SDQ | 6 years | Parent | 5,525 | 0.1 | (0.06, 0.14) | 9.58 x 10^-7^ |
| Peer problems prorated subscale of SDQ | 6 years | Parent | 5,521 | 0.08 | (0.04, 0.12) | 8.85 x 10^-5^ |
| Total prorated score of SDQ | 6 years | Parent | 5,510 | 0.37 | (0.24, 0.50) | 2.43 x 10^-8^ |
| Prosocial prorated subscale of SDQ | 8 years | Mother | 5,287 | -0.08 | (-0.14, -0.03) | 0.003 |
| Hyperactive prorated subscale of SDQ | 8 years | Mother | 5,282 | 0.12 | (0.05, 0.19) | 0.001 |
| Emotional prorated subscale of SDQ | 8 years | Mother | 5,282 | 0.17 | (0.12, 0.23) | 1.38 x 10^-9^ |
| Conduct prorated subscale of SDQ | 8 years | Mother | 5,283 | 0.12 | (0.08, 0.17) | 1.73 x 10^-7^ |
| Peer problems prorated subscale of SDQ | 8 years | Mother | 5,283 | 0.10 | (0.06, 0.14) | 1.33 x 10^-5^ |
| Total prorated score of SDQ | 8 years | Mother | 5,277 | 0.52 | (0.38, 0.67) | 2.08 x 10^-12^ |
| Prosocial prorated subscale of SDQ | 8 years | Teacher | 3,579 | -0.13 | (-0.21, -0.05) | 0.001 |
| Hyperactive prorated subscale of SDQ | 8 years | Teacher | 3,583 | 0.17 | (0.09, 0.26) | 8.85 x 10^-5^ |
| Emotional prorated subscale of SDQ | 8 years | Teacher | 3,583 | 0.14 | (0.07, 0.21) | 8.85 x 10^-5^ |
| Conduct prorated subscale of SDQ | 8 years | Teacher | 3,581 | 0.1 | (0.05, 0.14) | 1.33 x10^-5^ |
| Peer problems prorated subscale of SDQ | 8 years | Teacher | 3,583 | 0.1 | (0.04, 0.16) | 0.001 |
| Total prorated score of SDQ | 8 years | Teacher | 3,582 | 0.52 | (0.33, 0.71) | 8.13 x 10^-8^ |
| Prosocial prorated subscale of SDQ | 9 years | Parent | 5,545 | -0.04 | (-0.09, 0.00) | 0.073 |
| Hyperactive prorated subscale of SDQ | 9 years | Parent | 5,538 | 0.1 | (0.04, 0.16) | 0.001 |
| Emotional prorated subscale of SDQ | 9 years | Parent | 5,524 | 0.14 | (0.09, 0.19) | 4.07 x 10^-8^ |
| Conduct prorated subscale of SDQ | 9 years | Parent | 5,537 | 0.1 | (0.06, 0.14) | 9.58 x 10^-7^ |
| Peer problems prorated subscale of SDQ | 9 years | Parent | 5,532 | 0.1 | (0.06, 0.15) | 1.33 x 10^-5^ |
| Total prorated score of SDQ | 9 years | Parent | 5,516 | 0.45 | (0.31, 0.59) | 2.98 x 10^-10^ |
| Prosocial prorated subscale of SDQ | 11 years | Teacher | 4,270 | -0.06 | (-0.13, 0.02) | 0.128 |
| Hyperactive prorated subscale of SDQ | 11 years | Teacher | 4,270 | 0.09 | (0.02, 0.17) | 0.019 |
| Emotional prorated subscale of SDQ | 11 years | Teacher | 4,270 | 0.09 | (0.03, 0.15) | 0.002 |
| Conduct prorated subscale of SDQ | 11 years | Teacher | 4,269 | 0.08 | (0.03, 0.13) | 0.001 |
| Peer problems prorated subscale of SDQ | 11 years | Teacher | 4,270 | 0.07 | (0.02, 0.13) | 0.011 |
| Total prorated score of SDQ | 11 years | Teacher | 4,270 | 0.34 | (0.17, 0.51) | 8.85 x 10^-5^ |
| Prosocial prorated subscale of SDQ | 11 years | Parent | 5,132 | -0.02 | (-0.06, 0.03) | 0.507 |
| Hyperactive prorated subscale of SDQ | 11 years | Parent | 5,118 | 0.11 | (0.05, 0.17) | 0.001 |
| Emotional prorated subscale of SDQ | 11 years | Parent | 5,118 | 0.18 | (0.13, 0.23) | 1.71 x 10^-12^ |
| Conduct prorated subscale of SDQ | 11 years | Parent | 5,128 | 0.09 | (0.05, 0.13) | 1.03 x 10^-5^ |
| Peer problems prorated subscale of SDQ | 11 years | Parent | 5,130 | 0.1 | (0.05, 0.14) | 1.33 x 10^-5^ |
| Total prorated score of SDQ | 11 years | Parent | 5,128 | 0.47 | (0.33, 0.61) | 4.70 x 10^-11^ |
| Locus of control | 8 years | Child | 4,599 | 0.13 | (0.07, 0.19) | 2.17 x 10^-5^ |
| Self-esteem global score | 8 years | Child | 5,009 | -0.13 | (-0.24, -0.03) | 0.01 |
| Self-Esteem scholastic | 8 years | Child | 5,016 | -0.21 | (-0.31, -0.10) | 8.85 x 10^-5^ |
| IQ measured with WISC | 8 years | Child | 5,295 | -1.15 | (-1.61, -0.68) | 1.25 x 10^-6^ |

TableS7. Separate ordinal regressions of a child PRS for neuroticism on various emotional and behavioural outcomes.

| **Outcome variable**  **(DAWBA)** | **Age** | **Report** | **Number** | **OR** | **95% CIs** | **P-value** |
| --- | --- | --- | --- | --- | --- | --- |
| Attention deficit hyperactivity disorder (ADHD) | 7 years | Parent and teacher | 5,448 | 1.09 | (1.04, 1.16) | 0.004 |
| Conduct disorder | 7 years | Parent and teacher | 5,389 | 1.06 | (1.00, 1.13) | 0.036 |
| Depressive disorder | 7 years | Parent and teacher | 5,379 | 1.05 | (0.99, 1.12) | 0.074 |
| Generalised anxiety disorder (GAD) | 7 years | Parent and teacher | 5,446 | 1.13 | (1.06, 1.20) | 8.85 x 10^-5^ |
| Obsessive compulsive disorder (OCD) | 7 years | Parent and teacher | 5,463 | 1.08 | (1.00, 1.16) | 0.042 |
| Oppositional disorder | 7 years | Parent and teacher | 5,432 | 1.09 | (1.03, 1.15) | 0.002 |
| Separation anxiety | 7 years | Parent and teacher | 5,453 | 1.17 | (1.07, 1.27) | 0.0002 |
| Separation anxiety (ICD-10) | 7 years | Parent and teacher | 5,453 | 1.23 | (1.11, 1.38) | 0.0002 |
| Social phobia | 7 years | Parent and teacher | 5,456 | 1.06 | (1.00, 1.14) | 0.054 |
| Specific phobias | 7 years | Parent and teacher | 5,460 | 1.09 | (1.04, 1.16) | 0.001 |
| Any disorder | 7 years | Parent and teacher | 5,470 | 1.14 | (1.06, 1.21) | 8.85 x 10^-5^ |
| Any emotional disorder | 7 years | Parent and teacher | 5,470 | 1.14 | (1.07, 1.20) | 3.61 x 10^-6^ |
| Any anxiety disorder | 7 years | Parent and teacher | 5,470 | 1.13 | (1.07, 1.20) | 1.90 x 10^-5^ |
| Any behavioural disorder | 7 years | Parent and teacher | 5,432 | 1.09 | (1.04, 1.16) | 0.001 |
| Attention deficit hyperactivity disorder (ADHD) | 10 years | Parent | 5,336 | 1.11 | (1.04, 1.17) | 0.002 |
| Conduct disorder | 10 years | Parent | 5,279 | 1.03 | (0.97, 1.09) | 0.343 |
| Depressive disorder | 10 years | Parent | 5,264 | 1.11 | (1.04, 1.17) | 0.001 |
| Generalised anxiety disorder (GAD) | 10 years | Parent | 5,331 | 1.14 | (1.08, 1.21) | 3.61 x 10^-6^ |
| Obsessive compulsive disorder (OCD) | 10 years | Parent | 5,349 | 1.14 | (1.04, 1.24) | 0.002 |
| Oppositional disorder | 10 years | Parent | 5,323 | 1.09 | (1.04, 1.16) | 0.001 |
| Separation anxiety | 10 years | Parent | 5,157 | 1.21 | (1.11, 1.31) | 1.1 x 10^-5^ |
| Social phobia | 10 years | Parent | 5,344 | 1.12 | (1.04, 1.19) | 0.001 |
| Specific phobias | 10 years | Parent | 5,335 | 1.13 | (1.06, 1.20) | 8.85 x 10^-5^ |
| Any disorder | 10 years | Parent | 5,359 | 1.17 | (1.09, 1.25) | 1.40 x 10^-6^ |
| Any emotional disorder | 10 years | Parent | 5,358 | 1.16 | (1.09, 1.23) | 9.58 x 10^-7^ |
| Any anxiety disorder | 10 years | Parent | 5,358 | 1.19 | (1.12, 1.26) | 2.80 x 10^-8^ |
| Any behavioural disorder | 10 years | Parent | 5,323 | 1.09 | (1.03, 1.16) | 0.003 |

**Legend:** Ordinal regressions estimated with robust SEs adjusted for sex, age, and the first 5 principal components of genetic ancestry.

# Appendix S5. Results: Supplementary information on model selection and the selected linear mixed-effect model

### Fitted linear mixed models

We estimated five models: the first, did not contain child NEU PRS and characterised trajectories of internalising and externalising problems uniquely based on the age. Variance was allowed to change based on the scale employed. The second model included child NEU PRS, but not interaction terms among covariates were used. The third model included child NEU PRS and all the two-ways interactions hypothesised to be of interest in the study. The fourth model included both child NEU PRS and three-way interactions, and finally, the fifth model (estimated post-hoc) included four-ways interactions. The fourth model proved to be the best model to explain the data in our sample.

TableS8. Model selection criteria SDQ models

| Models | df | AIC | BIC | ICC individual level | ICC scale level |
| --- | --- | --- | --- | --- | --- |
| Model 1: No PRS all covariates | 13 | 163284.4 | 163406 | 0.2247954 | 0.4451464 |
| Model 2: Main association PRS | 19 | 107843.7 | 108014 | 0.2133103 | 0.4418742 |
| Model 3: All Two-ways interactions | 30 | 102238.1 | 102506.7 | 0.2272329 | 0.5379345 |
| Model 4: All Three-ways interactions | **36** | **102130.7** | **102452.9** | **0.2278699** | **0.5387127** |
| Model 5: All Four-ways interactions | 45 | 102208.9 | 102611.7 | 0.2278886 | 0.5386403 |

**Legend:** Model 1: mixed logtype_ age_8y i.scale_ i.scale_#c.age_8y reporter kz021_rc mat_ed mat_sclass || ID:age_8y, cov(un)|| scale_:

Model 2: mixed logtype_ c.child_3_std age_8y i.scale i.scale_#c.age_8y pc1_child-pc5_child reporter kz021_rc mat_ed mat_sclass || ID:age_8y , cov(un) || scale_:

Model 3: xtmixed logtype_ age_8y i.kz021_rc i.reporter i.scale_ child_3_std c.age_8y##c.child_3_std i.reporter##c.child_3_std scale_##c.child_3_std i.reporter##i.kz021_rc i.reporter##c.age_8y c.age_8y##i.kz021_rc i.scale_##i.kz021_rc i.scale_##c.age_8y i.scale_##i.reporter mat_ed mat_sclass c994_rc pc1_child-pc5_child || ID:age_8y, cov(un) || scale:reporter, cov(un) reml

Model 4: xtmixed logtype_ age_8y i.kz021_rc i.reporter i.scale_ child_3_std reporter##c.child_3_std scale_##c.child_3_std reporter##kz021_rc##c.age_8y i.scale_##i.kz021_rc##c.age_8y i.reporter##i.scale_##i.kz021_rc scale_##c.child_3_std##c.age_8y i.scale_##c.child_3_std##i.reporter c.age_8y##c.child_3_std##i.reporter mat_ed mat_sclass c994_rc pc1_child-pc5_child || ID:age_8y, cov(un) || scale:reporter, cov(un) reml

Model 5: xtmixed logtype_ age_8y i.kz021_rc i.reporter i.scale_ child_3_std i.scale_##i.kz021_rc##c.age_8y##i.reporter reporter##c.child_3_std##scale_##kz021_rc reporter##scale_##c.child_3_std##c.age_8y kz021_rc##scale_##c.child_3_std##c.age_8y mat_ed mat_sclass c994_rc pc1_child-pc5_child || ID:age_8y, cov(un) || scale:reporter, cov(un) reml

### Further information on the selected model

The median (IQR) age of the assessments where genetic information was available was 8.33 (6.75 – 10.83) years with a minimum age of 3.67 years and a maximum of 14.00 years (age was centred at its median). For these analyses, the natural log (plus 1) values of the outcomes were used for both analytical convenience (e.g., variance stabilisation and ensuring positivity on the outcome scale) as well as ease of interpretation (expressing covariate effects on the ratio scale, i.e., percentage change). Our analyses included all available data on the SDQ scale and the model implies that missing observations over time are missing at random (MAR), conditional on all included covariates. To mitigate the risk of bias due to attrition we included in these models additional covariates that were found to be strongly associated with attrition (i.e., maternal education, maternal social class, and maternal age).

We explored the role of the reporter (parent vs teacher) by including it both as a fixed as well as a random effect at the level representing the different scales used for each child. This permitted us to decompose the SDQ score variability further to that associated to different scales used and between reporters. This variance component was additional to between intra-child variability (level 2). To examine whether the child PRS for neuroticism was associated with the overall level of the trajectories, we included the PRS as a fixed-effect term and we used interaction terms to explore its effects depending on the scale used, the reporter of the scale, and the sex of the child. To examine how the PRS was associated with changes over time in our model, we included a main effect of the PRS and an interaction of the PRS with the fixed-effect age term.

### Random Effects findings

As reported in **Table 1,** at the individual child level we found strong evidence of between-child variation in both the overall levels of scores in addition to how these levels change over time. These variations were positively correlated which means that children with low scores were more stable over time while children with higher scores changed more over time. In the third level of this model, we also estimated the random variability at the scale level and its correlation with the reporter of the scale. Here, we found that the higher the difference in scores between scales (i.e., children predominantly scoring high on the externalising scale and low on the internalising scale) the lesser the impact of the reporter of the scale.

### Teacher reporting

Teacher reported generally less problems than parents regardless of the scale employed and the sex of the child. Specifically, teachers reported 11% (95% CI: 7 to 14%) fewer internalising problems in boys and rated girls 17% lower on the externalising scale (95% CI: 11 to 24%) than parents. Teachers reported more internalising problems as the child got older. Finally, the association of the child NEU PRS on both the overall levels of the trajectories and on the rate of change was weaker when the problems were teacher rated.

**Model diagnostics for selected mixed-effect model**

Here we report the graphical representations of the residual distributions to assess the linearity and normality assumptions. As the points in both graphs form a roughly straight line, we assumed that our model assumptions were met. See **FigS4** and **5**.

FigS4. Normal Probability Plot.

*
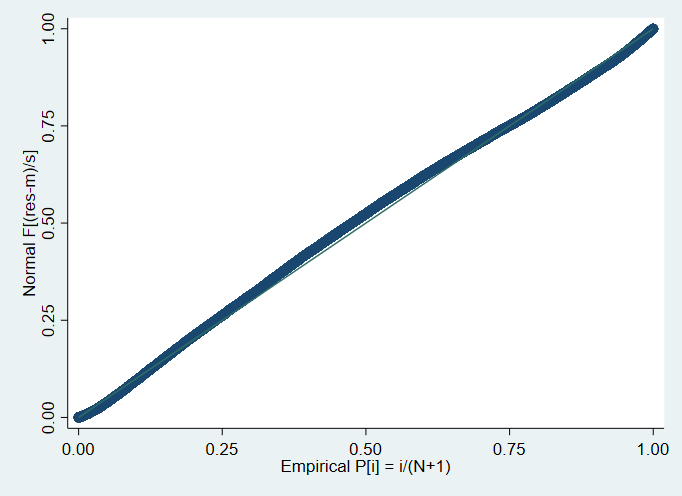
*

***Legend:*** *A Normal Probability Plot compares the values in a data set (on the y axis) with their associated quantile values derived from a standardised normal distribution (on the x axis). The normal probability plot is a graphical technique for normality testing; it aims to identify any relevant departures from normality.*

FigS5. Quantile-quantile (Q-Q) plot to assess normality of the residuals.

*
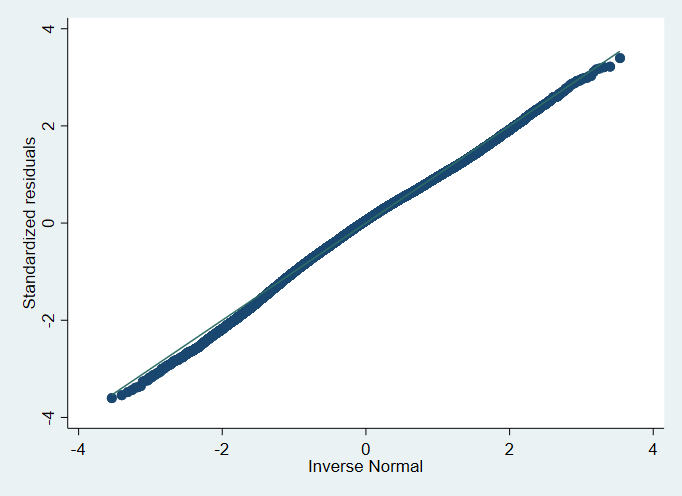
*

***Legend:*** *The Q-Q plot is a graphical tool used to evaluate whether the set of data has been plausibly derived from a normal distribution. By comparing how the dots sit on the straight line (indicating the theoretical quantiles indicating a normal distribution) it is possible to check if a normality assumption is plausible.*

FigS6. Trajectories of internalising and externalising problems by PRS levels across age when SDQ is teacher reported by sex of the child.


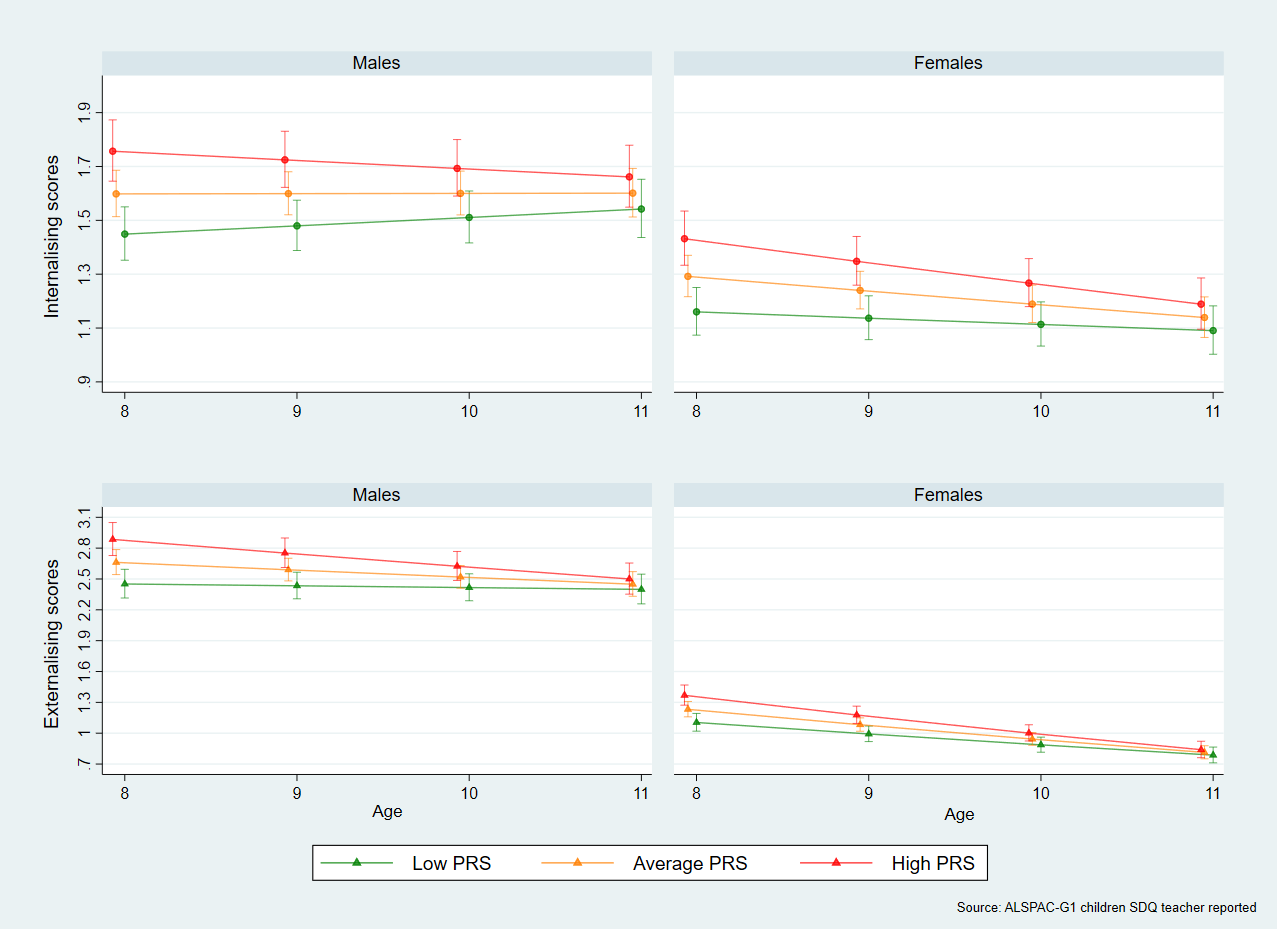


**Legend:** *Low PRS corresponds to 1SD lower than average score, Average PRS corresponds to the mean PRS score, and high PRS corresponds to 1 SD higher than average. Circles represent the internalising scale and the triangle the externalising.*

TableS9. Mean differences in low vs high child PRS at youngest compared to oldest child age by sex of the child and reporter.

| Mother reported | Youngest Age (4 years) | Oldest Age (11 years) | Mean difference youngest vs oldest |
| --- | --- | --- | --- |
| Females | **Mean (95% CI)** | **Mean (95% CI)** | **Mean (95% CI)** |
| Lowest PRS (SDQ Internalising) | 1.89 (1.81, 1.98) | 1.55 (1.47, 1.64) | 0.34 (0.34, 0.34) |
| Highest PRS (SDQ Internalising) | 2.15 (2.06, 2.24) | 1.94 (1.85, 2.04) | 0.21 (0.21, 0.20) |
| Mean Difference Low vs High PRS | -0.26 (-0.25, -0.26) | -0.39 (-0.38, 0.40) | 0.13 (0.13, 0.14) |
| Males | **Mean (95% CI)** | **Mean (95% CI)** | **Mean (95% CI)** |
| Lowest PRS (SDQ Internalising) | 2.04 (1.96, 2.12) | 1.47 (1.40, 1.55) | 0.57 (0.56, 0.57) |
| Highest PRS (SDQ Internalising) | 2.31 (2.22, 2.40) | 1.85 (1.76, 1.94) | 0.46 (0.46, 0.46) |
| Mean Difference Low vs High PRS | -0.27 (-0.26, -0.28) | -0.38 (-0.36, -0.39) | 0.11 (0.10, 0.11) |
| Females | **Mean (95% CI)** | **Mean (95% CI)** | **Mean (95% CI)** |
| Lowest PRS (SDQ Externalising) | 4.18 (4.04, 4.33) | 2.37 (2.27, 2.48) | 1.81 (1.77, 1.85) |
| Highest PRS (SDQ Externalising) | 4.48 (4.32, 4.64) | 2.63 (2.52, 2.75) | 1.85 (1.80, 1.89) |
| Mean Difference Low vs High PRS | -0.30 (-0.28, -0.31) | -0.26 (-0.25, -0.27) | -0.04 (-0.03, -0.04) |
| Males | **Mean (95% CI)** | **Mean (95% CI)** | **Mean (95% CI)** |
| Lowest PRS (SDQ Externalising) | 4.97 (4.80, 5.13) | 3.29 (3.16, 3.43) | 1.68 (1.64, 1.7) |
| Highest PRS (SDQ Externalising) | 5.31 (5.13, 5.49) | 3.62 (3.48, 3.77) | 1.69 (1.65, 1.72) |
| Mean Difference Low vs High PRS | -0.34 (-0.33, -0.36) | -0.33 (-0.32, -0.34) | -0.01 (-0.01, -0.02) |
| Teacher reported | **Youngest Age (8 years)** | **Oldest Age (11 years)** | **Mean difference Youngest vs Oldest** |
| Females | **Mean (95% CIs)** | **Mean (95% CI)** | **Mean (95% CI)** |
| Lowest PRS (SDQ Internalising) | 1.16 (1.07, 1.25) | 1.09 (1.00, 1.18) | 0.07 (0.07, 0.07) |
| Highest PRS (SDQ Internalising) | 1.14 (1.07, 1.22) | 1.19 (1.10, 1.29) | -0.05 (-0.03, -0.07) |
| Mean Difference Low vs High PRS | 0.02 (0.0, 0.03) | -0.10 (-0.10, -0.11) | -0.08 (-0.10, 0.14) |
| Males | **Mean (95% CIs)** | **Mean (95% CIs)** | **Mean (95% CIs)** |
| Lowest PRS (SDQ Internalising) | 1.45 (1.35, 1.55) | 1.54 (1.44, 1.65) | -0.09 (-0.09, -0.10) |
| Highest PRS (SDQ Internalising) | 1.76 (1.65, 1.87) | 1.66 (1.55, 1.78) | -0.10 (-0.10, 0.09) |
| Mean Difference Low vs High PRS | -0.31 (-0.30, -0.32) | -0.12 (-011, -0.13) | 0.19 (-0.19, -0.19) |
| Females | **Mean (95% CIs)** | **Mean (95% CIs)** | **Mean (95% CIs)** |
| Lowest PRS (SDQ Externalising) | 1.10 (1.02, 1.19) | 0.79 (0.71, 0.87) | 0.31 (0.31, 0.32) |
| Highest PRS (SDQ Externalising) | 1.37 (1.27, 1.47) | 0.84 (0.76, 0.92) | 0.53 (0.51, 0.55) |
| Mean Difference Low vs High PRS | -0.27 (-0.25, -0.28) | -0.05 (-0.05, -0.05) | -0.22 (0.20, -0.23) |
| Males | **Mean (95% CIs)** | **Mean (95% CIs)** | **Mean (95% CIs)** |
| Lowest PRS (SDQ Externalising) | 2.45 (2.31, 2.59) | 2.40 (2.26, 2.55) | 0.05 (0.05, 0.04) |
| Highest PRS (SDQ Externalising) | 2.89 (2.73, 3.05) | 2.50 (2.35, 2.65) | 0.39 (0.38, 0.40) |
| Mean Difference Low vs High PRS | -0.44 (-0.42, -0.46) | -0.10 (-0.09, -0.10) | -0.34 (-0.33, -0.36) |

# Appendix S6. Results: Sensitivity analyses to explore potential differential misclassification of the outcome.

Whilst testing for potential differential misclassification of the child outcomes, we found little evidence of an association between maternal NEU PRS and child’s behavioural and emotional problems as rated by the teacher, as assessed by 95% confidence intervals overlapping with the null (**TableS10**). The maternal NEU PRS had little or no influence on teachers’ assessment of child’s behaviours once accounting for the child’s own PRS, minimising the likelihood that mothers with a higher NEU PRS may have influenced the teacher’s opinion of the child (e.g., through their behaviours during scholastic meetings). Second, while we found that mothers and teachers did indeed rate child’s emotional and behavioural problems differently (mean of the difference in score ≠ 0) (**TableS11**), with mothers rating the child’s behavioural and emotional problems higher than teachers, when we examined whether maternal PRS was associated with a “difference score”, we found little evidence to support this hypothesis. In addition, effect estimates were small and with differing directions of effect, suggesting that the association of the maternal PRS with “difference in score” may be negligible in our sample (**TableS12**).

Here, we speculate on a few potential mechanisms. First, differences in scores due to reporters (mother vs teacher) may reflect actual differences in behaviour of children across different situational contexts (i.e., cross-situational differences) (Achenbach et al., 1987), where the child manifests certain behaviours and emotional reactions with higher frequency and/or intensity in a familiar context than in a structured environment such as school. Second, differences in scores may relate to different abilities of parents and teachers in recognising psychological problems of children. In fact, parents may be more sensitive to small fluctuations in mood than teachers who need to pay attention to multiple children simultaneously. Third, parents and children share their genetics; this could open other pathways through which child’s emotional and behavioural problems are manifested differently when in the presence of the parent. In fact, parents may be evoking different behavioural and emotional responses in children both via direct (i.e., genetic confounding) and indirect (e.g., parenting practices) genetic effects (Warrington et al., 2021). For example, a mother or a father with a high NEU PRS may use more discipline and/or harsher parenting practices which may, in turn, evoke more conduct problems or more withdrawal reactions in the child. In this scenario, a parental PRS would be acting as a confounding factor and parenting practices would be on the causal pathway as mediators of the association.

TableS10. Associations between maternal neuroticism PRS and child’s emotional and behavioural difficulties as rated by the teacher adjusted for the child’s neuroticism PRS.

| **Outcome** | **Number** | **Beta** | **95% CIs** | **P-values** |
| --- | --- | --- | --- | --- |
| SDQ_prosoc_8y | 2,389 | -0.05 | (-0.15,0.06) | 0.375 |
| SDQ_hyperac_8y | 2,392 | 0.06 | (-0.06,0.17) | 0.312 |
| SDQ_emot_8y | 2,392 | 0.03 | (-0.06,0.12) | 0.533 |
| SDQ_cond_8y | 2,391 | -0.00 | (-0.06,0.06) | 0.963 |
| SDQ_peer_8y | 2,392 | 0.02 | (-0.07,0.10) | 0.715 |
| SDQ_total_8y | 2,392 | 0.10 | (-0.16,0.35) | 0.456 |
| SDQ_prosoc_11y | 2,841 | -0.04 | (-0.14,0.05) | 0.358 |
| SDQ_hyperac_11y | 2,841 | 0.02 | (-0.08,0.12) | 0.693 |
| SDQ_emot_11y | 2,841 | 0.04 | (-0.04,0.11) | 0.314 |
| SDQ_cond_11y | 2,841 | -0.01 | (-0.07,0.05) | 0.800 |
| SDQ_peer_11y | 2,841 | 0.03 | (-0.05,0.11) | 0.448 |
| SDQ_total_11y | 2,841 | 0.08 | (-0.15,0.31) | 0.487 |

**Legend:** All outcomes are reported by the teacher. Separate linear regression with robust SEs were employed. Analyses were adjusted for child’s sex, age, PRS, and the first five principal components of genetic ancestry.

TableS11. Descriptives of “difference in score” variables.

| **Variable** | **Number** | **Mean** | **SD** | **Min** | **Max** |
| --- | --- | --- | --- | --- | --- |
| Prosocial difference score (teacher 8y – mother 8y) | 3,685 | -0.20 | 2.64 | -9 | 8 |
| Hyperactivity difference score (teacher 8y – mother 8y) | 3,682 | -0.93 | 2.60 | -10 | 9 |
| Emotional difference score (teacher 8y – mother 8y) | 3,683 | -0.38 | 2.22 | -9 | 9 |
| Conduct difference score (teacher 8y – mother 8y) | 3,683 | -0.84 | 1.56 | -8 | 8 |
| Peer problems difference score (teacher 8y – mother 8y) | 3,684 | -0.19 | 1.83 | -9 | 10 |
| Total difference score (teacher 8y – mother 8y) | 3,678 | -2.35 | 5.50 | -28 | 21 |
| Prosocial difference score (teacher 11y – mother 11y) | 4,144 | -0.25 | 2.54 | -9 | 10 |
| Hyperactivity difference score (teacher 11y – mother 11y) | 4,123 | -0.81 | 2.47 | -10 | 9 |
| Emotional difference score (teacher 11y – mother 11y) | 4,132 | -0.28 | 2.15 | -9 | 9 |
| Conduct difference score (teacher 11y – mother 11y) | 4,143 | -0.56 | 1.62 | -9 | 9 |
| Peer problems difference score (teacher 11y – mother 11y) | 4,147 | -0.00 | 1.84 | -9 | 10 |
| Total difference score (teacher 11y – mother 11y) | 4,138 | -1.64 | 5.46 | -29 | 25 |

**Legend:** Child’s scores on the SDQ subscales and total score as rated by the mother were subtracted from the child’s score on the SDQ as rated by the teacher at similar ages. Negative means indicate higher SDQ scores as rated by the mother.

TableS12. Associations between maternal neuroticism PRS and “difference in score” outcomes (teacher - mother reported scores) unadjusted and adjusted for child neuroticism PRS.

|  |  | **Unadjusted complete case** | | **Adjusted for child PRS** | |
| --- | --- | --- | --- | --- | --- |
| **Outcome (difference in scores)** | **Number** | **Beta** | **95% CIs** | **Beta** | **95% CIs** |
| Prosocial (teacher 8y – mother 8y) | 1,805 | -0.05 | (-0.17,0.07) | -0.10 | (-0.24,0.04) |
| Hyperactivity (teacher 8y – mother 8y) | 1,805 | 0.06 | (-0.06,0.18) | -0.03 | (-0.17,0.10) |
| Emotional (teacher 8y – mother 8y) | 1,807 | 0.06 | (-0.04,0.16) | 0.10 | (-0.01,0.22) |
| Conduct (teacher 8y – mother 8y) | 1,806 | 0.03 | (-0.04,0.10) | 0.04 | (-0.04,0.13) |
| Peer problems (teacher 8y – mother 8y) | 1,807 | 0.05 | (-0.03,0.14) | 0.08 | (-0.02,0.18) |
| Total score (teacher 8y – mother 8y) | 1,804 | 0.19 | (-0.06,0.44) | 0.29 | (0.00,0.58) |
| Prosocial (teacher 11y – mother 11y) | 2,104 | 0.02 | (-0.08,0.13) | -0.02 | (-0.14,0.10) |
| Hyperactivity (teacher 11y – mother 11y) | 2,096 | -0.09 | (-0.19,0.01) | -0.11 | (-0.22,0.01) |
| Emotional (teacher 11y – mother 11y) | 2,098 | -0.08 | (-0.16,0.01) | -0.04 | (-0.14,0.06) |
| Conduct (teacher 11y – mother 11y) | 2,105 | -0.06 | (-0.12,0.01) | -0.06 | (-0.14,0.02) |
| Peer problems (teacher 11y – mother 11y) | 2,104 | -0.03 | (-0.10,0.05) | 0.01 | (-0.08,0.10) |
| Total score (teacher 11y – mother 11y) | 2,102 | -0.25 | (-0.48,-0.03) | -0.20 | (-0.46,0.06) |

**Legend:** Separate linear regression with robust SEs were employed. Analyses were adjusted for child’s sex, age, PRS, and the first five principal components of genetic ancestry. SDQ subscales and total score at 8 and 11 years of age when reported by both mothers and teachers.

# Appendix S7. Results: Sensitivity analyses: exploring the causal nature of primary analyses

To address one of the secondary aims of this study (i.e., estimating the causal relationship between the child neuroticism PRS and later emotional and behavioural difficulties) we adjusted our analyses for the maternal PRS for neuroticism in the separate multivariable linear regressions in order to account for a possible confounding effect of this variable. Paternal PRS was not employed in models for two main reasons: first, we lacked statistical power as only 1,711 fathers had genetic information available for this analysis. Second, as suggested in prior studies (Kong et al., 2018), maternal and paternal genetics may have differential effects on the child’s phenotype. Here, we hypothesised that the greatest variance in child’s genotype and phenotype would have been contributed by maternal genotype via a greater “genetic nurturing” effect.

### Sensitivity analyses investigating the potential causal nature of the association between child NEU PRS and later psychological difficulties

To estimate a potential causal association between the child PRS and later emotional and behavioural problems we adjusted for the maternal NEU PRS (i.e., a potential confounder of this association). Our effect estimates remained consistent across most of the results (**TableS13)**. However, incorporating the maternal PRS into the model led to larger confidence intervals around our effect estimates, reducing their precision.

# Appendix S8. Results: Sensitivity analyses: Addressing missing data

### Sensitivity analyses investigating the potential bias arising from attrition

Results obtained in the 100 multiply imputed datasets are presented in the **Appendix S9**. Monte Carlo errors were <10% of the standard error and fraction of missing information (FMI) values were no larger than 11 in the Carey models imputation and 54 in the SDQ model imputation, both indicating an adequate level of statistical reproducibility of the multiply imputed analyses (Madley-Dowd et al., 2019). Findings obtained in the multiply imputed datasets were largely consistent (with the most different result observed for the total score of the SDQ at 9 years: N complete case analysis (cca)= 5,516, β_cca_: 0.45, 95% CI_cca_: (0.31 to 0.59), N imputed (i)= 7,451, β_i_: 0.50, 95% CI_i_: (0.37 to 0.63) with those reported in the CCA, suggesting that the levels of attrition did not substantially bias our effect estimates under the assumption that data were MAR.

|  | Mother’s educational attainment (n=12,482) | | Maternal social class (10,109) | | Maternal age at pregnancy (12,060) | | Mother’s post-partum EPDS score (12,150) | | Maternal PRS to neuroticism  (7,826) | | Child PRS to neuroticism  (7,851) | |
| --- | --- | --- | --- | --- | --- | --- | --- | --- | --- | --- | --- | --- |
| **Models** | **OR** | **95% CIs** | **OR** | **95% CIs** | **OR** | **95% CIs** | **OR** | **95% CIs** | **OR** | **95% CIs** | **OR** | **95% CIs** |
| Carey_6m Model^a^ | 0.65 | (0.61,0.68) | 1.52 | (1.40,1.65) | 0.93 | (0.93,0.94) | 1.05 | (1.04,1.05) | 1.07 | (1.03,1.12) | 1.10 | (1.04,1.16) |
| Carey_24m Model^a^ | 0.62 | (0.59,0.65) | 1.58 | (1.46,1.72) | 0.93 | (0.92,0.93) | 1.04 | (1.04,1.05) | 1.08 | (1.03,1.13) | 1.09 | (1.03,1.15) |
| SDQ_4y Model^b^ | 0.64 | (0.60,0.67) | 1.57 | (1.44,1.70) | 0.93 | (0.92,0.94) | 1.05 | (1.04,1.05) | 1.06 | (1.02,1.11) | 1.07 | (1.02,1.12) |
| SDQ_6y Model^b^ | 0.61 | (0.58,0.64) | 1.65 | (1.52,1.79) | 0.92 | (0.92,0.93) | 1.05 | (1.04,1.05) | 1.09 | (1.04,1.14) | 1.07 | (1.02,1.12) |
| SDQ_8y Model^b^ | 0.58 | (0.55,0.61) | 1.74 | (1.60,1.88) | 0.92 | (0.91,0.93) | 1.05 | (1.04,1.05) | 1.06 | (1.01,1.11) | 1.07 | (1.02,1.12) |
| SDQ_8y* Model^b^ | 0.79 | (0.75,0.84) | 1.22 | (1.12,1.33) | 0.96 | (0.95,0.97) | 1.03 | (1.02,1.04) | 1.01 | (0.96,1.06) | 1.03 | (0.98,1.07) |
| SDQ_9y Model^b^ | 0.59 | (0.56,0.62) | 1.78 | (1.64,1.93) | 0.92 | (0.91,0.93) | 1.05 | (1.04,1.05) | 1.05 | (1.00,1.09) | 1.07 | (1.01,1.12) |
| SDQ_11y* Model^b^ | 0.85 | (0.81,0.89) | 1.16 | (1.06,1.26) | 0.97 | (0.96,0.98) | 1.03 | (1.02,1.03) | 1.02 | (0.98,1.07) | 0.99 | (0.95,1.03) |
| SDQ_11y Model^b^ | 0.58 | (0.55,0.61) | 1.71 | (1.57,1.85) | 0.92 | (0.92,0.93) | 1.05 | (1.04,1.06) | 1.07 | (1.02,1.12) | 1.08 | (1.03,1.13) |
| Dawba_7y Model^c^ | 0.59 | (0.57,0.63) | 1.65 | (1.52,1.78) | 0.92 | (0.92,0.93) | 1.05 | (1.04,1.05) | 1.07 | (1.02,1.11) | 1.08 | (1.03,1.13) |
| Dawba_10y Model^c^ | 0.59 | (0.56,0.62) | 1.71 | (1.57,1.85) | 0.92 | (0.92,0.93) | 1.05 | (1.04,1.06) | 1.07 | (1.02,1.12) | 1.10 | (1.05,1.15) |

TableS13. OR for missing data in the main outcomes’ models.

**Legend:** The models with the asterisk* represent those models where the reporter of the child emotional and behavioural problem was the teacher. Mother’s educational attainment (three levels; highest level corresponds to highest education), Maternal social class (dichotomous variable; higher levels corresponding to lower social class), Mother smoked in pregnancy (dichotomous variable (No/Yes) indicating women who smoked during the first three months of pregnancy, Mother’s post-partum depression (continuous score on EPDS for mothers of the index child). a: all Carey subscales and covariates used in the models (i.e., child’s sex, child PRS for neuroticism and the first five principal components of genetic ancestry);

b: all SDQ subscales and covariates used in the models (i.e., child’s sex, child’s age, child PRS to neuroticism and the first five principal components of genetic ancestry;

c: all DAWBA scales reported and covariates used in the models (i.e., child’s sex, child’s age, child PRS to neuroticism and the first five principal components of genetic ancestry).

TableS14. Associations between child PRS for neuroticism and various psychological outcomes in multiple imputed datasets.

|  |  |  | **Analyses in complete dataset** | | | | **Analysis in multiply imputed datasets** | | | |
| --- | --- | --- | --- | --- | --- | --- | --- | --- | --- | --- |
| **Outcome variable (Linear regression models)** | **Age** | **Reporter** | **Number** | **Beta** | **95% CIs** | **P-values** | **Number** | **Beta** | **95% CIs** | **P-values** |
| *Activity | 6 months | Mother | 6,271 | -0.16 | (-0.33, 0.00) | 0.057 | 6,871 | -0.16 | (-0.33,0.00) | 0.06 |
| *Rhythm | 6 months | Mother | 6,269 | 0.08 | (-0.10, 0.26) | 0.393 | 6,871 | 0.08 | (-0.10,0.27) | 0.37 |
| Approach | 6 months | Mother | 6,266 | 0.18 | (0.01, 0.35) | 0.034 | 6,871 | 0.18 | (0.02, 0.035) | 0.03 |
| *Adaptability subscale | 6 months | Mother | 6,271 | 0.11 | (-0.04, 0.27) | 0.139 | 6,871 | 0.11 | (-0.04,0.26) | 0.15 |
| *Intensity | 6 months | Mother | 6,269 | 0.01 | (-0.14, 0.16) | 0.896 | 6,871 | 0.01 | (-0.14,0.16) | 0.91 |
| *Mood | 6 months | Mother | 6,267 | 0.03 | (-0.12, 0.19) | 0.665 | 6,871 | 0.03 | (-0.12,0.18) | 0.70 |
| *Persistence subscale | 6 months | Mother | 6,269 | -0.03 | (-0.16, 0.11) | 0.716 | 6,871 | -0.03 | (-0.16,0.11) | 0.72 |
| *Distract | 6 months | Mother | 6,271 | 0.07 | (-0.07, 0.22) | 0.323 | 6,871 | 0.07 | (-0.07,0.22) | 0.32 |
| *Threshold | 6 months | Mother | 6,264 | 0.21 | (0.05, 0.37) | 0.010 | 6,871 | 0.21 | (0.05,0.37) | 0.01 |
| *Activity | 24 months | Mother | 6,260 | 0.08 | (-0.04, 0.20) | 0.174 | 6,871 | 0.09 | (-0.03, 0.21) | 0.16 |
| *Rhythm | 24 months | Mother | 6,257 | 0.25 | (0.09, 0.40) | 0.002 | 6,871 | 0.27 | (0.12, 0.42) | 0.001 |
| *Approach subscale | 24 months | Mother | 6,253 | 0.17 | (-0.03, 0.36) | 0.092 | 6,871 | 0.18 | (-0.02, 0.39) | 0.07 |
| *Adaptability subscale | 24 months | Mother | 6,233 | 0.15 | (0.04, 0.27) | 0.006 | 6,871 | 0.17 | (0.07, 0.28) | 0.002 |
| *Intensity | 24 months | Mother | 6,255 | 0.14 | (0.02, 0.26) | 0.027 | 6,871 | 0.15 | (0.03, 0.27) | 0.02 |
| *Mood | 24 months | Mother | 6,259 | 0.21 | (0.06, 0.36) | 0.005 | 6,871 | 0.24 | (0.09, 0.39) | 0.001 |
| *Persistence subscale | 24 months | Mother | 6,252 | 0.20 | (0.07, 0.32) | 0.002 | 6,871 | 0.20 | (0.08, 0.33) | 0.002 |
| *Distract | 24 months | Mother | 6,255 | 0.07 | (-0.05, 0.20) | 0.260 | 6,871 | 0.07 | (-0.06, 0.19) | 0.29 |
| *Threshold | 24 months | Mother | 6,260 | -0.10 | (-0.21, 0.02) | 0.113 | 6,871 | -0.09 | (-0.02, 0.02) | 0.11 |
| Total score of SDQ | 4 years | Mother | 5,788 | 0.27 | (0.15, 0.40) | 2.30 x 10^-5^ | 7,451 | 0.30 | (0.18, 0.42) | 9.58 x 10^-7^ |
| Total score of SDQ | 6 years | Mother | 5,510 | 0.37 | (0.24, 0.50) | 2.43 x 10-8 | 7,451 | 0.39 | (0.27, 0.52) | 9.64 x 10^-10^ |
| Total score of SDQ | 8 years | Mother | 5,277 | 0.52 | (0.38, 0.67) | 2.08 x 10^-12^ | 7,451 | 0.53 | (0.39, 0.67) | 1.17 x 10^-13^ |
| Total score of SDQ | 8 years | Teacher | 3,582 | 0.52 | (0.33, 0.71) | 8.13 x 10^-8^ | 7,451 | 0.53 | (0.35, 0.70) | 2.92 x 10^-9^ |
| Total score of SDQ | 9 years | Mother | 5,516 | 0.45 | (0.31, 0.59) | 2.98 x 10^-10^ | 7,451 | 0.50 | (0.37, 0.63) | 4.76 x 10^-14^ |
| Total score of SDQ | 11 years | Mother | 5,128 | 0.47 | (0.33, 0.61) | 4.70 x 10^-11^ | 7,451 | 0.48 | (0.35, 0.62) | 3.19 x 10^-12^ |
| Total score of SDQ | 11 years | Teacher | 4,270 | 0.34 | (0.17, 0.51) | 8.85 x 10^-5^ | 7,451 | 0.32 | (0.15, 0.49) | 0.0002 |

**Legend**: Separate linear regressions with robust SEs adjusted for child’s sex, age, and the first five principal components of genetic ancestry. *Subscales of the prorated score of the Carey Infant Temperament Scales (CTSs). The N in the imputed data refers to the number of participants who had the genetic information (NEU PRS).

TableS15. Associations between neuroticism PRS and all psychological outcomes, unadjusted and adjusted for maternal neuroticism PRS.

|  | | | | **Complete Unadjusted for maternal PRS** | | | **Complete adjusted for maternal PRS** | | |
| --- | --- | --- | --- | --- | --- | --- | --- | --- | --- |
| **Outcome variable (Linear regression models)** | **Age** | **Report** | **Number** | **Beta** | **95% CIs** | **P-values** | **Beta** | **95% CIs** | **P-values** |
| Prorated score of Carey – Activity subscale | 6 months | Mother | 4,271 | -0.12 | (-0.32,0.09) | 0.27 | -0.12 | (-0.35,0.11) | 0.30 |
| Prorated score of Carey – Rhythm subscale | 6 months | Mother | 4,271 | 0.04 | (-0.19,0.26) | 0.76 | 0.04 | (-0.21,0.29) | 0.78 |
| Prorated score of Carey – Approach subscale | 6 months | Mother | 4,268 | 0.25 | (0.05,0.45) | 0.02 | 0.27 | (0.05,0.50) | 0.02 |
| Prorated score of Carey – Adaptability subscale | 6 months | Mother | 4,272 | 0.18 | (-0.00,0.36) | 0.06 | 0.27 | (0.07,0.47) | 0.01 |
| Prorated score of Carey – Intensity subscale | 6 months | Mother | 4,270 | 0.09 | (-0.10,0.27) | 0.35 | 0.07 | (-0.14,0.28) | 0.51 |
| Prorated score of Carey – Mood subscale | 6 months | Mother | 4,269 | 0.08 | (-0.10,0.27) | 0.380 | 0.18 | (-0.04,0.39) | 0.105 |
| Prorated score of Carey – Persistence subscale | 6 months | Mother | 4,270 | -0.05 | (-0.21,0.11) | 0.55 | 0.07 | (-0.12,0.25) | 0.47 |
| Prorated score of Carey – Distract subscale | 6 months | Mother | 4,271 | 0.15 | (-0.03,0.33) | 0.10 | 0.20 | (0.00,0.40) | 0.05 |
| Prorated score of Carey – Threshold | 6 months | Mother | 4,269 | 0.22 | (0.03,0.41) | 0.02 | 0.15 | (-0.06,0.37) | 0.16 |
| Prorated score of Carey – Activity subscale | 24 months | Mother | 4,241 | 0.15 | (0.01,0.29) | 0.03 | 0.16 | (0.00,0.32) | 0.05 |
| Prorated score of Carey – Rhythm subscale | 24 months | Mother | 4,238 | 0.31 | (0.12,0.49) | 2.276 x 10^-8^ | 0.26 | (0.04,0.47) | 0.02 |
| Prorated score of Carey – Approach subscale | 24 months | Mother | 4,235 | 0.10 | (-0.14,0.35) | 0.40 | 0.02 | (-0.26,0.30) | 0.89 |
| Prorated score of Carey – Adaptability subscale | 24 months | Mother | 4,229 | 0.19 | (0.06,0.33) | 0.01 | 0.17 | (0.02,0.32) | 0.03 |
| Prorated score of Carey – Intensity subscale | 24 months | Mother | 4,237 | 0.18 | (0.03,0.33) | 0.02 | 0.20 | (0.02,0.37) | 0.03 |
| Prorated score of Carey – Mood subscale | 24 months | Mother | 4,241 | 0.29 | (0.11,0.47) | 2.276 x 10^-8^ | 0.30 | (0.09,0.50) | 0.004 |
| Prorated score of Carey – Persistence subscale | 24 months | Mother | 4,235 | 0.21 | (0.05,0.36) | 0.01 | 0.22 | (0.04,0.40 | 0.02 |
| Prorated score of Carey – Distract subscale | 24 months | Mother | 4,238 | 0.04 | (-0.11,0.19) | 0.60 | 0.07 | (-0.10,0.24) | 0.44 |
| Prorated score of Carey – Threshold | 24 months | Mother | 4,241 | -0.10 | (-0.25,0.04) | 0.17 | -0.15 | (-0.31,0.02) | 0.08 |
| Prosocial prorated subscale of SDQ | 4 years | Mother | 3,990 | -0.06 | (-0.12,0.01) | 0.08 | -0.04 | (-0.12,0.03) | 0.24 |
| Hyperactive prorated subscale of SDQ | 4 years | Mother | 3,996 | 0.09 | (0.01,0.16) | 0.02 | 0.06 | (-0.03,0.14) | 0.18 |
| Emotional prorated subscale of SDQ | 4 years | Mother | 3,996 | 0.08 | (0.03,0.13) | 0.003 | 0.07 | (0.01,0.12) | 0.02 |
| Conduct prorated subscale of SDQ | 4 years | Mother | 3,990 | 0.10 | (0.05,0.14) | 2.276 x 10^-8^ | 0.07 | (0.02,0.13) | 0.01 |
| Peer problems prorated subscale of SDQ | 4 years | Mother | 3,994 | 0.09 | (0.04,0.14) | 0.0004 | 0.05 | (-0.00,0.11) | 0.06 |
| Total prorated score of SDQ | 4 years | Mother | 3,980 | 0.35 | (0.20,0.50) | 1.327 x 10^-5^ | 0.25 | (0.08,0.42) | 0.003 |
| Prosocial prorated subscale of SDQ | 6 years | Mother | 3,805 | -0.02 | (-0.08,0.03) | 0.41 | -0.05 | (-0.11,0.02) | 0.14 |
| Hyperactive prorated subscale of SDQ | 6 years | Mother | 3,802 | 0.07 | (-0.01,0.15) | 0.07 | 0.04 | (-0.05,0.13) | 0.35 |
| Emotional prorated subscale of SDQ | 6 years | Mother | 3,801 | 0.12 | (0.07,0.17) | 2.55 x 10^-6^ | 0.10 | (0.04,0.17) | 0.001 |
| Conduct prorated subscale of SDQ | 6 years | Mother | 3,804 | 0.09 | (0.04,0.14) | 0.0004 | 0.09 | (0.03,0.14) | 0.002 |
| Peer problems prorated subscale of SDQ | 6 years | Mother | 3,801 | 0.08 | (0.03,0.12) | 0.001 | 0.07 | (0.02,0.13) | 0.006 |
| Total prorated score of SDQ | 6 years | Mother | 3,796 | 0.34 | (0.19,0.50) | 1.71 x 10^-5^ | 0.29 | (0.11,0.47) | 0.002 |
| Prosocial prorated subscale of SDQ | 8 years | Mother | 3,668 | -0.08 | (-0.14, -0.01) | 0.02 | -0.07 | (-0.14, -0.00) | 0.05 |
| Hyperactive prorated subscale of SDQ | 8 years | Mother | 3,665 | 0.11 | (0.03,0.20) | 0.01 | 0.08 | (-0.02,0.17) | 0.11 |
| Emotional prorated subscale of SDQ | 8 years | Mother | 3,666 | 0.19 | (0.13,0.25) | 5.41 x 10^-10^ | 0.17 | (0.10,0.25) | 8.89 x 10^-6^ |
| Conduct prorated subscale of SDQ | 8 years | Mother | 3,666 | 0.12 | (0.07,0.17) | 2.55 x 10^-6^ | 0.11 | (0.05,0.17) | 0.0003 |
| Peer problems prorated subscale of SDQ | 8 years | Mother | 3,666 | 0.10 | (0.05,0.15) | 8.86 x 10^-5^ | 0.09 | (0.03,0.15) | 0.003 |
| Total prorated score of SDQ | 8 years | Mother | 3,663 | 0.53 | (0.36,0.71) | 2.92 x 10^-9^ | 0.45 | (0.25,0.66) | 1.69 x 10^-5^ |
| Prosocial prorated subscale of SDQ | 8 years | Teacher | 2,389 | -0.12 | (-0.22, -0.02) | 0.02 | -0.10 | (-0.21,0.01) | 0.09 |
| Hyperactive prorated subscale of SDQ | 8 years | Teacher | 2,392 | 0.14 | (0.03,0.24) | 0.01 | 0.11 | (-0.01,0.23) | 0.08 |
| Emotional prorated subscale of SDQ | 8 years | Teacher | 2,392 | 0.13 | (0.05,0.21) | 0.003 | 0.12 | (0.02,0.21) | 0.02 |
| Conduct prorated subscale of SDQ | 8 years | Teacher | 2,391 | 0.09 | (0.04,0.14) | 0.001 | 0.09 | (0.03,0.15) | 0.01 |
| Peer problems prorated subscale of SDQ | 8 years | Teacher | 2,392 | 0.11 | (0.04,0.18) | 0.002 | 0.10 | (0.02,0.19) | 0.02 |
| Total prorated score of SDQ | 8 years | Teacher | 2,392 | 0.47 | (0.25,0.70) | 4.24 x 10^-5^ | 0.42 | (0.16,0.69) | 0.002 |
| Prosocial prorated subscale of SDQ | 9 years | Mother | 3,811 | -0.05 | (-0.10,0.01) | 0.09 | -0.05 | (-0.11,0.01) | 0.13 |
| Hyperactive prorated subscale of SDQ | 9 years | Mother | 3,805 | 0.09 | (0.02,0.16) | 0.02 | 0.05 | (-0.04,0.13) | 0.26 |
| Emotional prorated subscale of SDQ | 9 years | Mother | 3,802 | 0.13 | (0.07,0.19) | 2.17 x 10^-5^ | 0.09 | (0.03,0.16) | 0.004 |
| Conduct prorated subscale of SDQ | 9 years | Mother | 3,806 | 0.09 | (0.04,0.14) | 0.0004 | 0.07 | (0.02,0.12) | 0.01 |
| Peer problems prorated subscale of SDQ | 9 years | Mother | 3,802 | 0.12 | (0.07,0.17) | 2.55 x 10^-6^ | 0.09 | (0.03,0.15) | 0.002 |
| Total prorated score of SDQ | 9 years | Mother | 3,795 | 0.43 | (0.27,0.59) | 1.38 x 10^-7^ | 0.30 | (0.12,0.49) | 0.002 |
| Prosocial prorated subscale of SDQ | 11 years | Teacher | 2,841 | -0.05 | (-0.14,0.03) | 0.24 | -0.03 | (-0.13,0.07) | 0.54 |
| Hyperactive prorated subscale of SDQ | 11 years | Teacher | 2,841 | 0.09 | (-0.00,0.19) | 0.06 | 0.08 | (-0.03,0.19) | 0.14 |
| Emotional prorated subscale of SDQ | 11 years | Teacher | 2,841 | 0.07 | (0.00,0.14) | 0.04 | 0.05 | (-0.02,0.13) | 0.17 |
| Conduct prorated subscale of SDQ | 11 years | Teacher | 2,841 | 0.06 | (0.01,0.12) | 0.03 | 0.07 | (0.00,0.13) | 0.04 |
| Peer problems prorated subscale of SDQ | 11 years | Teacher | 2,841 | 0.05 | (-0.02,0.12) | 0.19 | 0.03 | (-0.05,0.11) | 0.44 |
| Total prorated score of SDQ | 11 years | Teacher | 2,841 | 0.27 | (0.06,0.49) | 0.01 | 0.23 | (-0.01,0.48) | 0.06 |
| Prosocial prorated subscale of SDQ | 11 years | Mother | 3,621 | -0.04 | (-0.09,0.02) | 0.20 | -0.03 | (-0.09,0.04) | 0.42 |
| Hyperactive prorated subscale of SDQ | 11 years | Mother | 3,613 | 0.88 | (0.74,1.02) | 7.07 x 10^-35^ | 0.04 | (-0.05,0.12) | 0.37 |
| Emotional prorated subscale of SDQ | 11 years | Mother | 3,614 | 0.18 | (0.12,0.23) | 1.41 x 10^-10^ | 0.15 | (0.08,0.22) | 2.67 x 10^-5^ |
| Conduct prorated subscale of SDQ | 11 years | Mother | 3,622 | 0.06 | (0.01,0.11) | 0.02 | 0.03 | (-0.02,0.09) | 0.24 |
| Peer problems prorated subscale of SDQ | 11 years | Mother | 3,622 | 0.10 | (0.04,0.15) | 0.0004 | 0.08 | (0.02,0.14) | 0.009 |
| Total prorated score of SDQ | 11 years | Mother | 3,620 | 0.40 | (0.23,0.57) | 3.99 x 10^-6^ | 0.29 | (0.10,0.49) | 0.003 |
| Locus of control | 8 years | Child | 3,796 | 0.16 | (0.05,0.27) | 0.004 | 0.17 | (0.05,0.30) | 0.007 |
| Self-esteem global score | 8 years | Child | 3,796 | -0.15 | (-0.37,0.07) | 0.18 | -0.09 | (-0.34,0.17) | 0.50 |
| Self-Esteem scholastic | 8 years | Child | 3,796 | -0.21 | (-0.42, -0.01) | 0.04 | -0.14 | (-0.37,0.10) | 0.26 |
| IQ measured with WISC | 8 years | Child | 3,796 | -1.12 | (-1.85, -0.39) | 0.003 | -1.15 | (-1.97, -0.33) | 0.006 |
| **Outcome variable (Ordinal regression models)** | **Age** | **Report** | **Number** | **LogOdds** | **95% CIs** | **P-values** | **LogOdds** | **95% CIs** | **P-values** |
| Attention deficit hyperactivity disorder (ADHD) | 7 years | Mother and teacher | 3,799 | 0.08 | (0.01,0.15) | 0.02 | 0.05 | (-0.03,0.13) | 0.23 |
| Hyperkinesis | 7 years | Mother and teacher | 3,799 | 0.09 | (0.02,0.16) | 0.02 | 0.05 | (-0.03,0.13) | 0.21 |
| Conduct disorder | 7 years | Mother and teacher | 3,755 | 0.05 | (-0.02,0.12) | 0.15 | 0.03 | (-0.05,0.11) | 0.46 |
| Depressive disorder | 7 years | Mother and teacher | 3,751 | 0.07 | (-0.00,0.14) | 0.06 | 0.05 | (-0.03,0.13) | 0.26 |
| Generalised anxiety disorder (GAD) | 7 years | Mother and teacher | 3,797 | 0.12 | (0.05,0.18) | 0.001 | 0.10 | (0.02,0.18) | 0.01 |
| Obsessive compulsive disorder (OCD) | 7 years | Mother and teacher | 3,809 | 0.09 | (-0.00,0.17) | 0.06 | 0.02 | (-0.08,0.13) | 0.63 |
| Oppositional disorder | 7 years | Mother and teacher | 3,789 | 0.09 | (0.02,0.15) | 0.01 | 0.07 | (-0.01,0.15) | 0.08 |
| Separation anxiety | 7 years | Mother and teacher | 3,802 | 0.14 | (0.04,0.25) | 0.01 | 0.14 | (0.02,0.25) | 0.03 |
| Separation anxiety (ICD-10) | 7 years | Mother and teacher | 3,802 | 0.24 | (0.10,0.38) | 0.001 | 0.21 | (0.05,0.38) | 0.01 |
| Social phobia | 7 years | Mother and teacher | 3,804 | 0.11 | (0.03,0.18) | 0.01 | 0.13 | (0.04,0.21) | 0.004 |
| Specific phobias | 7 years | Mother and teacher | 3,809 | 0.10 | (0.03,0.16) | 0.01 | 0.10 | (0.03,0.18) | 0.01 |
| Any disorder | 7 years | Mother and teacher | 3,815 | 0.12 | (0.05,0.20) | 0.002 | 0.12 | (0.03,0.20) | 0.01 |
| Any emotional disorder | 7 years | Mother and teacher | 3,815 | 0.13 | (0.06,0.20) | 0.002 | 0.13 | (0.06,0.21) | 0.001 |
| Any anxiety disorder | 7 years | Mother and teacher | 3,815 | 0.12 | (0.05,0.19) | 0.001 | 0.12 | (0.04,0.20) | 0.002 |
| Any behavioural disorder | 7 years | Mother and teacher | 3,789 | 0.09 | (0.02,0.16) | 0.01 | 0.08 | (0.00,0.16) | 0.05 |
| Attention deficit hyperactivity disorder (ADHD) | 10 years | Mother | 3,726 | 0.08 | (0.00,0.15) | 0.04 | 0.04 | (-0.04,0.12) | 0.32 |
| Conduct disorder | 10 years | Mother | 3,694 | 0.01 | (-0.06,0.08) | 0.83 | 0.01 | (-0.07,0.09) | 0.84 |
| Depressive disorder | 10 years | Mother | 3,686 | 0.05 | (-0.02,0.13) | 0.13 | 0.03 | (-0.05,0.11) | 0.45 |
| Generalised anxiety disorder (GAD) | 10 years | Mother | 3,723 | 0.12 | (0.05,0.19) | 0.001 | 0.11 | (0.03,0.19) | 0.01 |
| Obsessive compulsive disorder (OCD) | 10 years | Mother | 3,734 | 0.14 | (0.04,0.24) | 0.005 | 0.13 | (0.03,0.24) | 0.02 |
| Oppositional disorder | 10 years | Mother | 3,720 | 0.08 | (0.01,0.15) | 0.02 | 0.05 | (-0.03,0.13) | 0.21 |
| Separation anxiety | 10 years | Mother | 3,597 | 0.22 | (0.12,0.32) | 1.62 x 10^-5^ | 0.22 | (0.11,0.34) | 3.27 x 10^-11^ |
| Social phobia | 10 years | Mother | 3,731 | 0.11 | (0.03,0.18) | 0.006 | 0.10 | (0.01,0.18) | 0.03 |
| Specific phobias | 10 years | Mother | 3,724 | 0.15 | (0.08,0.22) | 2.67 x 10^-5^ | 0.13 | (0.05,0.21) | 0.001 |
| Any disorder | 10 years | Mother | 3,740 | 0.14 | (0.07,0.22) | 0.0003 | 0.11 | (0.02,0.20) | 0.02 |
| Any emotional disorder | 10 years | Mother | 3,740 | 0.16 | (0.09,0.23) | 7.46 x 10^-6^ | 0.15 | (0.07,0.23) | 0.0002 |
| Any anxiety disorder | 10 years | Mother | 3,740 | 0.18 | (0.11,0.25) | 4.66 x 10^-7^ | 0.17 | (0.09,0.25) | 3.11 x 10^-5^ |
| Any behavioural disorder | 10 years | Mother | 3,720 | 0.08 | (0.01,0.15) | 0.02 | 0.06 | (-0.02,0.14) | 0.15 |

**Legend:** Separate linear or ordinal regressions with robust SEs adjusted for child’s sex, age, the first 5 principal components of genetic ancestry and maternal PRS for neuroticism.

# Appendix S9. Results: Sensitivity analyses: Using genome-wide significant p-value threshold NEU PRS

### Sensitivity analyses investigating the specificity of the association between child NEU PRS and the psychological outcomes via neuroticism and not via other pathways (i.e., minimising pleiotropy).

We re-performed our single-level analyses using a PRS constructed using a genome-wide significant P-value threshold with and without adjustment for the maternal NEU PRS. The findings attenuated across all SDQ scales with the exception of the emotional subscale once using a PRS with a GWAS p-value threshold, suggesting that the observed association between child NEU PRS and the conduct and behavioural subscales may be driven by underlying symptoms of anxiety (**TableS14**). Very weak evidence was found at 4 years of age for an association between the child NEU PRS and the total scores of emotional and behavioural difficulties (β=0.05, 95% CI: -0.14 to 0.24). However, as early as at 6 years, we found some evidence of an association between the child neuroticism PRS and the total scores of emotional and behavioural difficulties (β=0.22, 95% CI: 0.02 to 0.42). We also found evidence for an association between the child NEU PRS and a higher risk of separation anxiety, both when assessed using the DSM-IV and ICD-10 criteria (OR: 1.21, 95% CI: 1.05 to 1.37; OR: 1.39, 95% CI: 1.17 to 1.62), social phobia (OR: 1.17, 95% CI: 1.05 to 1.29), and specific phobia (OR: 1.16, 95% CI: 1.05 to 1.27). These results remained largely consistent upon adjusting for the maternal NEU PRS.

The betas are shown for a genome-wide significant (P < 5 × 10^-8^) PRS created from 113 SNPs in ALSPAC children. On the left side of the table, results from complete case analyses of the child PRS using a P < 5 × 10^-8^ threshold limited to participants with maternal genotype available but unadjusted for maternal PRS are presented. On the right side of the table, results from analyses using a child PRS at a P < 5 × 10^-8^ threshold and adjusted for maternal PRS for neuroticism are presented. Both models were adjusted for the same covariates as described in the main methods section.

TableS16. Associations between child PRS for neuroticism (threshold P < 5 × 10-8) and all psychological outcomes unadjusted and adjusted for maternal PRS for neuroticism.

|  | | | | **Complete unadjusted for maternal PRS** | | | **Complete adjusted for maternal PRS** | | |
| --- | --- | --- | --- | --- | --- | --- | --- | --- | --- |
| **Outcome variable (Linear regression models)** | **Age** | **Report** | **Number** | **Beta** | **95% CIs** | **P-values** | **Beta** | **95% CIs** | **P-values** |
| Prorated score of Carey – Activity subscale | 6 months | Mother | 4,271 | 0.14 | (-0.17,0.46) | 0.369 | 0.15 | (-0.16,0.47) | 0.336 |
| Prorated score of Carey – Rhythm subscale | 6 months | Mother | 4,271 | 0.24 | (-0.11,0.59) | 0.186 | 0.24 | (-0.12,0.59) | 0.187 |
| Prorated score of Carey – Approach subscale | 6 months | Mother | 4,268 | 0.23 | (-0.07,0.53) | 0.132 | 0.22 | (-0.08,0.52) | 0.154 |
| Prorated score of Carey – Adaptability subscale | 6 months | Mother | 4,272 | 0.22 | (-0.06,0.49) | 0.127 | 0.23 | (-0.05,0.51) | 0.102 |
| Prorated score of Carey – Intensity subscale | 6 months | Mother | 4,270 | 0.08 | (-0.21,0.36) | 0.595 | 0.06 | (-0.22,0.35) | 0.657 |
| Prorated score of Carey – Mood subscale | 6 months | Mother | 4,269 | 0.01 | (-0.27,0.30) | 0.924 | 0.04 | (-0.25,0.32) | 0.805 |
| Prorated score of Carey – Persistence subscale | 6 months | Mother | 4,270 | -0.07 | (-0.32,0.18) | 0.588 | -0.03 | (-0.28,0.23) | 0.826 |
| Prorated score of Carey – Distract subscale | 6 months | Mother | 4,271 | 0.02 | (-0.25,0.28) | 0.905 | 0.02 | (-0.25,0.29) | 0.880 |
| Prorated score of Carey – Threshold | 6 months | Mother | 4,269 | -0.13 | (-0.42,0.17) | 0.411 | -0.17 | (-0.47,0.13) | 0.271 |
| Prorated score of Carey – Activity subscale | 24 months | Mother | 4,241 | 0.13 | (-0.09,0.35) | 0.260 | 0.12 | (-0.11,0.34) | 0.304 |
| Prorated score of Carey – Rhythm subscale | 24 months | Mother | 4,238 | 0.50 | (0.21,0.79) | 0.001 | 0.46 | (0.17,0.75) | 0.002 |
| Prorated score of Carey – Approach subscale | 24 months | Mother | 4,235 | 0.003 | (-0.38,0.39) | 0.987 | -0.03 | (-0.42,0.36) | 0.871 |
| Prorated score of Carey – Adaptability subscale | 24 months | Mother | 4,229 | 0.10 | (-0.11,0.31) | 0.346 | 0.08 | (-0.13,0.28) | 0.477 |
| Prorated score of Carey – Intensity subscale | 24 months | Mother | 4,237 | 0.11 | (-0.11,0.34) | 0.326 | 0.11 | (-0.13,0.34) | 0.368 |
| Prorated score of Carey – Mood subscale | 24 months | Mother | 4,241 | 0.08 | (-0.19,0.36) | 0.558 | 0.06 | (-0.22,0.34) | 0.673 |
| Prorated score of Carey – Persistence subscale | 24 months | Mother | 4,235 | 0.13 | (-0.12,0.37) | 0.306 | 0.11 | (-0.13,0.36) | 0.354 |
| Prorated score of Carey – Distract subscale | 24 months | Mother | 4,238 | -0.05 | (-0.28,0.19) | 0.696 | -0.04 | (-0.28,0.19) | 0.721 |
| Prorated score of Carey – Threshold | 24 months | Mother | 4,241 | 0.16 | (-0.06,0.39) | 0.158 | 0.16 | (-0.07,0.39) | 0.166 |
| Prosocial prorated subscale of SDQ | 4 years | Mother | 3,990 | -0.03 | (-0.12,0.07) | 0.613 | -0.02 | (-0.12,0.08) | 0.731 |
| Hyperactive prorated subscale of SDQ | 4 years | Mother | 3,996 | 0.03 | (-0.09,0.14) | 0.664 | 0.01 | (-0.11,0.13) | 0.859 |
| Emotional prorated subscale of SDQ | 4 years | Mother | 3,996 | 0.10 | (0.02,0.18) | 0.012 | 0.09 | (0.01,0.17) | 0.022 |
| Conduct prorated subscale of SDQ | 4 years | Mother | 3,990 | 0.01 | (-0.06,0.08) | 0.726 | -0.002 | (-0.07,0.07) | 0.954 |
| Peer problems prorated subscale of SDQ | 4 years | Mother | 3,994 | 0.03 | (-0.04,0.11) | 0.406 | 0.01 | (-0.06,0.09) | 0.732 |
| Total prorated score of SDQ | 4 years | Mother | 4,061 | 0.16 | (-0.07,0.39) | 0.176 | 0.10 | (-0.13,0.34) | 0.386 |
| Prosocial prorated subscale of SDQ | 6 years | Mother | 3,805 | -0.07 | (-0.16,0.02) | 0.142 | -0.07 | (-0.16,0.02) | 0.112 |
| Hyperactive prorated subscale of SDQ | 6 years | Mother | 3,802 | 0.03 | (-0.10,0.15) | 0.649 | 0.02 | (-0.11,0.14) | 0.815 |
| Emotional prorated subscale of SDQ | 6 years | Mother | 3,801 | 0.13 | (0.05,0.22) | 0.003 | 0.12 | (0.03,0.21) | 0.008 |
| Conduct prorated subscale of SDQ | 6 years | Mother | 3,804 | 0.08 | (0.01,0.16) | 0.031 | 0.08 | (-0.00,0.15) | 0.054 |
| Peer problems prorated subscale of SDQ | 6 years | Mother | 3,801 | 0.10 | (0.03,0.17) | 0.008 | 0.09 | (0.02,0.16) | 0.014 |
| Total prorated score of SDQ | 6 years | Mother | 3,796 | 0.35; | (0.11,0.60) | 0.005 | 0.31 | (0.06,0.56) | 0.013 |
| Prosocial prorated subscale of SDQ | 8 years | Mother | 3,668 | -0.07 | (-0.17,0.03) | 0.148 | -0.07 | (0.17,0.03) | 0.196 |
| Hyperactive prorated subscale of SDQ | 8 years | Mother | 3,665 | 0.05 | (-0.08,0.18) | 0.450 | 0.03 | (-0.10,0.16) | 0.660 |
| Emotional prorated subscale of SDQ | 8 years | Mother | 3,666 | 0.13; | (0.03,0.22) | 0.008 | 0.11 | (0.01,0.21) | 0.025 |
| Conduct prorated subscale of SDQ | 8 years | Mother | 3,666 | 0.10 | (0.02,0.17) | 0.010 | 0.08 | (0.01,0.16) | 0.030 |
| Peer problems prorated subscale of SDQ | 8 years | Mother | 3,666 | 0.06 | (-0.02,0.15) | 0.132 | 0.05 | (-0.03,0.14) | 0.221 |
| Total prorated score of SDQ | 8 years | Mother | 3,663 | 0.35 | (0.08,0.62) | 0.012 | 0.28 | (0.01,0.55) | 0.045 |
| Prosocial prorated subscale of SDQ | 8 years | Teacher | 2,389 | -0.05 | (-0.20,0.10) | 0.538 | -0.03 | (-0.18,0.12) | 0.700 |
| Hyperactive prorated subscale of SDQ | 8 years | Teacher | 2,392 | 0.002 | (-0.16,0.16) | 0.976 | -0.02 | (-0.18,0.14) | 0.814 |
| Emotional prorated subscale of SDQ | 8 years | Teacher | 2,392 | 0.14 | (0.01,0.26) | 0.031 | 0.12 | (-0.003,0.25) | 0.055 |
| Conduct prorated subscale of SDQ | 8 years | Teacher | 2,391 | 0.08 | (-0.00,0.17) | 0.059 | 0.07 | (-0.01,0.16) | 0.087 |
| Peer problems prorated subscale of SDQ | 8 years | Teacher | 2,392 | 0.05 | (-0.06,0.15) | 0.398 | 0.03 | (-0.07,0.14) | 0.532 |
| Total prorated score of SDQ | 8 years | Teacher | 2,392 | 0.27 | (-0.06,0.61) | 0.110 | 0.22 | (-0.12,0.56) | 0.204 |
| Prosocial prorated subscale of SDQ | 9 years | Mother | 3,811 | -0.04 | (-0.13,0.04) | 0.334 | -0.04 | (-0.12,0.05) | 0.383 |
| Hyperactive prorated subscale of SDQ | 9 years | Mother | 3,805 | 0.02 | (-0.09,0.14) | 0.712 | 0.002 | (-0.12,0.12) | 0.976 |
| Emotional prorated subscale of SDQ | 9 years | Mother | 3,802 | 0.13 | (0.04,0.21) | 0.005 | 0.11 | (0.02,0.19) | 0.018 |
| Conduct prorated subscale of SDQ | 9 years | Mother | 3,806 | 0.02 | (-0.05,0.09) | 0.586 | 0.005 | (-0.07,0.08) | 0.892 |
| Peer problems prorated subscale of SDQ | 9 years | Mother | 3,802 | -0.002 | (-0.08,0.08) | 0.956 | -0.02 | (-0.10,0.06) | 0.580 |
| Total prorated score of SDQ | 9 years | Mother | 3,795 | 0.17 | (-0.08,0.42) | 0.188 | 0.09 | (-0.16,0.34) | 0.481 |
| Prosocial prorated subscale of SDQ | 11 years | Teacher | 2,841 | -0.06 | (-0.19,0.08) | 0.408 | -0.05 | (-0.19,0.09) | 0.502 |
| Hyperactive prorated subscale of SDQ | 11 years | Teacher | 2,841 | 0.09 | (-0.06,0.25) | 0.249 | 0.08 | (-0.08,0.24) | 0.307 |
| Emotional prorated subscale of SDQ | 11 years | Teacher | 2,841 | 0.11 | (0.01,0.22) | 0.035 | 0.10 | (-0.00,0.21) | 0.057 |
| Conduct prorated subscale of SDQ | 11 years | Teacher | 2,841 | 0.04 | (-0.05,0.13) | 0.396 | 0.04 | (-0.06,0.13) | 0.446 |
| Peer problems prorated subscale of SDQ | 11 years | Teacher | 2,841 | 0.04 | (-0.07,0.15) | 0.476 | 0.03 | (-0.08,0.14) | 0.576 |
| Total prorated score of SDQ | 11 years | Teacher | 2,841 | 0.29 | (-0.06,0.63) | 0.102 | 0.25 | (-0.09,0.60) | 0.150 |
| Prosocial prorated subscale of SDQ | 11 years | Mother | 3,621 | -0.01 | (-0.10,0.08) | 0.795 | -0.01 | (-0.10,0.09) | 0.904 |
| Hyperactive prorated subscale of SDQ | 11 years | Mother | 3,613 | 0.05 | (-0.07,0.17) | 0.421 | 0.03 | (-0.09,0.15) | 0.612 |
| Emotional prorated subscale of SDQ | 11 years | Mother | 3,614 | 0.10 | (0.01,0.20) | 0.032 | 0.08 | (-0.01,0.17) | 0.095 |
| Conduct prorated subscale of SDQ | 11 years | Mother | 3,622 | 0.03 | (-0.05,0.10) | 0.471 | 0.02 | (-0.06,0.09) | 0.691 |
| Peer problems prorated subscale of SDQ | 11 years | Mother | 3,622 | 0.05 | (-0.04,0.13) | 0.272 | 0.03 | (-0.05,0.12) | 0.429 |
| Total prorated score of SDQ | 11 years | Mother | 3,620 | 0.23 | (-0.04,0.50) | 0.088 | 0.17 | (-0.11,0.44) | 0.230 |
| Locus of control | 8 years | Child | 3,796 | 0.08 | (-0.09,0.25) | 0.343 | 0.07 | (-0.10,0.25) | 0.403 |
| Self-esteem global score | 8 years | Child | 3,796 | -0.14 | (-0.50,0.21) | 0.422 | -0.11 | (-0.47,0.24) | 0.526 |
| Self-Esteem scholastic | 8 years | Child | 3,796 | -0.17 | (-0.49,0.16) | 0.307 | -0.13 | (-0.45,0.20) | 0.439 |
| IQ measured with WISC | 8 years | Child | 3,796 | -0.58 | (-1.68,0.52) | 0.299 | -0.50 | (-1.61,0.60) | 0.373 |
| **Outcome variable (Ordinal regression models)** | **Age** | **Report** | **Number** | **LogOdds** | **Confidence Intervals** | **P-values** | **LogOdds** | **Confidence Intervals** | **P-values** |
| Attention deficit hyperactivity disorder (ADHD) | 7 years | Mother and teacher | 3,799 | 0.02 | (-0.09,0.14) | 0.670 | 0.01 | (-0.11,0.12) | 0.914 |
| Hyperkinesis | 7 years | Mother and teacher | 3,799 | 0.03 | (-0.08,0.14) | 0.606 | 0.01 | (-0.10,0.12) | 0.847 |
| Conduct disorder | 7 years | Mother and teacher | 3,755 | 0.003 | (-0.11,0.11) | 0.955 | -0.01 | (-0.12,0.10) | 0.882 |
| Depressive disorder | 7 years | Mother and teacher | 3,751 | 0.05 | (-0.05,0.16) | 0.331 | 0.04 | (-0.07,0.15) | 0.449 |
| Generalised anxiety disorder (GAD) | 7 years | Mother and teacher | 3,797 | 0.02 | (-0.09,0.12) | 0.720 | 0.004 | (-0.10,0.11) | 0.936 |
| Obsessive compulsive disorder (OCD) | 7 years | Mother and teacher | 3,809 | 0.10 | (-0.05,0.25) | 0.175 | 0.08 | (-0.07,0.23) | 0.310 |
| Oppositional disorder | 7 years | Mother and teacher | 3,789 | 0.09 | (-0.01,0.20) | 0.087 | 0.08 | (-0.03,0.19) | 0.136 |
| Separation anxiety | 7 years | Mother and teacher | 3,802 | 0.21 | (0.05,0.37) | 0.012 | 0.20 | (0.03,0.36) | 0.019 |
| Separation anxiety (ICD-10) | 7 years | Mother and teacher | 3,802 | 0.39 | (0.17,0.62) | 0.001 | 0.37 | (0.14,0.59) | 0.002 |
| Social phobia | 7 years | Mother and teacher | 3,804 | 0.17 | (0.05,0.29) | 0.004 | 0.17 | (0.05,0.29) | 0.005 |
| Specific phobias | 7 years | Mother and teacher | 3,809 | 0.16 | (0.05,0.27) | 0.003 | 0.16 | (0.05,0.26) | 0.004 |
| Any disorder | 7 years | Mother and teacher | 3,815 | 0.08 | (-0.03,0.20) | 0.150 | 0.07 | (0.04,0.19) | 0.225 |
| Any emotional disorder | 7 years | Mother and teacher | 3,815 | 0.07 | (-0.03,0.17) | 0.157 | 0.06 | (-0.04,0.17) | 0.221 |
| Any anxiety disorder | 7 years | Mother and teacher | 3,815 | 0.07 | (-0.03,0.18) | 0.161 | 0.06 | (-0.04,0.17) | 0.239 |
| Any behavioural disorder | 7 years | Mother and teacher | 3,789 | 0.08 | (-0.04,0.19) | 0.180 | 0.07 | (-0.05,0.18) | 0.243 |
| Attention deficit hyperactivity disorder (ADHD) | 10 years | Mother | 3,726 | 0.002 | (-0.11,0.12) | 0.973 | -0.02 | (-0.13,0.10) | 0.783 |
| Conduct disorder | 10 years | Mother | 3,694 | 0.12 | (0.00,0.23) | 0.050 | 0.12 | (0.00,0.23) | 0.049 |
| Depressive disorder | 10 years | Mother | 3,686 | 0.04 | (-0.07,0.15) | 0.507 | 0.03 | (-0.09,0.14) | 0.656 |
| Generalised anxiety disorder (GAD) | 10 years | Mother | 3,723 | 0.07 | (-0.03,0.18) | 0.173 | 0.06 | (-0.05,0.17) | 0.260 |
| Obsessive compulsive disorder (OCD) | 10 years | Mother | 3,734 | -0.02 | (-0.18,0.14) | 0.789 | -0.04 | (-0.19,0.12) | 0.652 |
| Oppositional disorder | 10 years | Mother | 3,720 | 0.08 | (-0.03,0.18) | 0.169 | 0.06 | (-0.05,0.17) | 0.287 |
| Separation anxiety | 10 years | Mother | 3,597 | 0.12 | (-0.04,0.28) | 0.134 | 0.11 | (-0.06,0.27) | 0.199 |
| Social phobia | 10 years | Mother | 3,731 | 0.14 | (0.02,0.26) | 0.023 | 0.13 | (0.01,0.25) | 0.039 |
| Specific phobias | 10 years | Mother | 3,724 | 0.09 | (-0.02,0.20) | 0.107 | 0.07 | (-0.04,0.18) | 0.206 |
| Any disorder | 10 years | Mother | 3,740 | 0.12 | (0.001,0.23) | 0.048 | 0.10 | (-0.02,0.21) | 0.109 |
| Any emotional disorder | 10 years | Mother | 3,740 | 0.09 | (-0.01,0.20) | 0.090 | 0.07 | (-0.03,0.18) | 0.173 |
| Any anxiety disorder | 10 years | Mother | 3,740 | 0.18 | (0.11,0.25) | 4.66 x 10^-7^ | 0.06 | (-0.05,0.17) | 0.266 |
| Any behavioural disorder | 10 years | Mother | 3,720 | 0.08 | (-0.03,0.19) | 0.150 | 0.07 | (-0.04,0.18) | 0.231 |

**Legend:** Separate linear and ordinal regression with robust SEs were employed. “Unadjusted” analyses were adjusted for child’s sex, age, and the first five principal components of genetic ancestry. “Adjusted” analyses included child’s sex, age, the first five principal components of genetic ancestry and the maternal PRS for neuroticism as covariates.

**Appendix S10: Discussion**

**Additional details on alternative explanations as to why parents and teachers’ scores of child emotional and behavioural problems differ: focus on genetic confounding.**

Parents and children share their genetics; this could open other pathways through which child’s emotional and behavioural problems are manifested differently when in the presence of the parent. In fact, parents may be evoking different behavioural and emotional responses in children both via direct (i.e., genetic confounding) and indirect (e.g., parenting practices) genetic effects (Warrington et al., 2021). For example, a mother or a father with a high PRS for neuroticism may use more discipline and/or harsher parenting practices which may, in turn, evoke more conduct problems or more withdrawal reactions in the child. In this scenario, a parental PRS would be acting as a confounding factor and parenting practices would be on the causal pathway as mediators of the association (**FigS7**). Dysfunctional parenting practices could reflect both underlying mental health problems (e.g., anxiety and depression) and/or a direct response to a child’s behaviour. Similarly, a child PRS as expressed via temperamental difficulties and/or more dysfunctional behaviours may have an evocative effect on parents, eliciting worse parenting practices or poorer mental health in the parents, which could reinforce a dysfunctional cycle. Nevertheless, exploring evocative genetic effects is beyond the scope of this study.

FigS7. Directed Acyclic Graph (DAG) representing potential pathways through which children behave/experience emotional symptoms differently in presence of a parent.


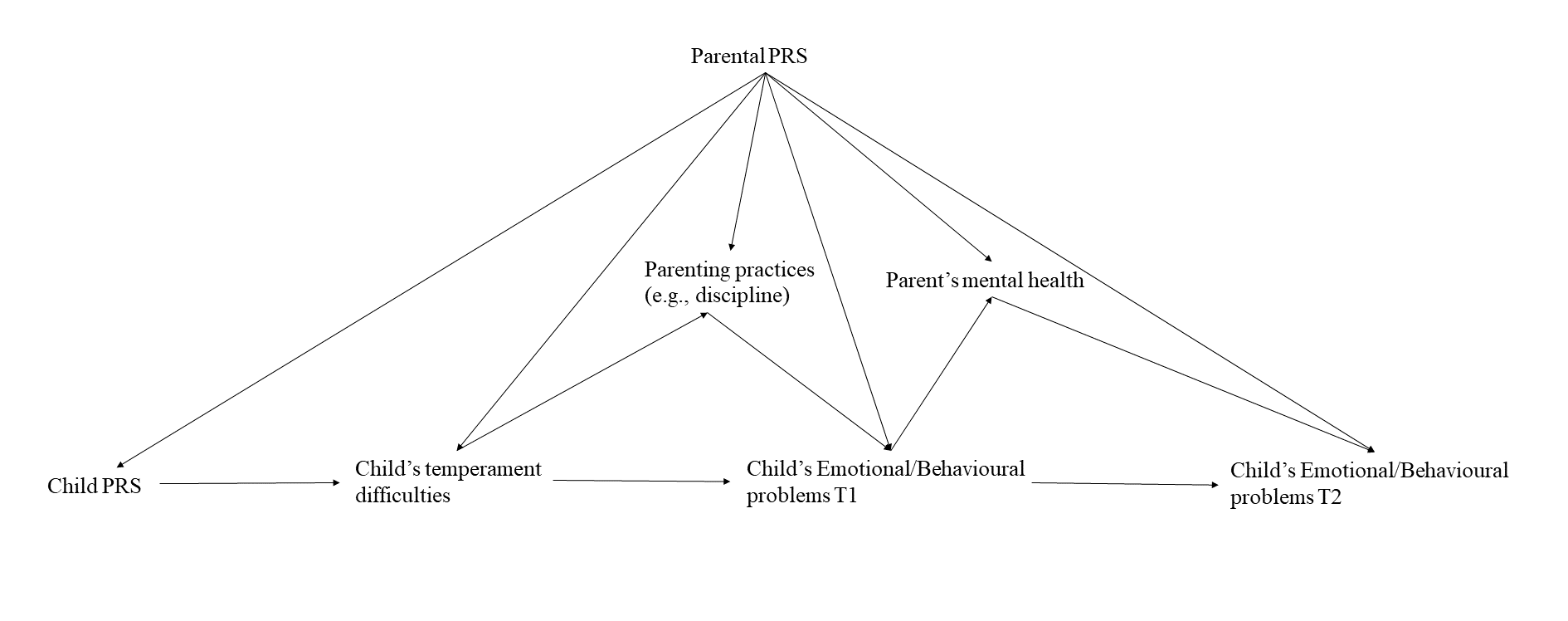


**Legend:** This DAG illustrates a hypothesised causal pathway from a child PRS for neuroticism to temperamental, emotional and behavioural problems and its relationship with parental genotype, parental parenting practices, and parental mental health. Dysfunctional parenting practices could reflect both underlying mental health problems (e.g., anxiety and depression) and/or a direct response to a child’s behaviour. Similarly, a child PRS as expressed via temperamental difficulties and/or more dysfunctional behaviours may have an evocative effect on parents, eliciting worse parenting practices or poorer mental health in the parents, which could reinforce a dysfunctional cycle.

# References

Achenbach, T. M., McConaughy, S. H., & Howell, C. T. (1987). Child/adolescent behavioral and emotional problems: implications of cross-informant correlations for situational specificity. *Psychological Bulletin*, *101*(2), 213.

Chess, S., & Thomas, A. (2013). *Temperament: Theory and practice*. Routledge.

Goodman, A., Heiervang, E., Collishaw, S., & Goodman, R. (2011). The ‘DAWBA bands’ as an ordered-categorical measure of child mental health: description and validation in British and Norwegian samples. *Social Psychiatry and Psychiatric Epidemiology*, *46*(6), 521–532.

Harter, S. (1985). *Manual for the self-perception profile for children:(revision of the perceived competence scale for children)*. University of Denver.

Hughes, R. A., Heron, J., Sterne, J. A. C., & Tilling, K. (2019). Accounting for missing data in statistical analyses: multiple imputation is not always the answer. *International Journal of Epidemiology*, *48*(4), 1294–1304.

Kong, A., Thorleifsson, G., Frigge, M. L., Vilhjalmsson, B. J., Young, A. I., Thorgeirsson, T. E., Benonisdottir, S., Oddsson, A., Halldorsson, B. V, Masson, G., Gudbjartsson, D. F., Helgason, A., Bjornsdottir, G., Thorsteinsdottir, U., & Stefansson, K. (2018). The nature of nurture: Effects of parental genotypes. *Science*, *359*(6374), 424 LP – 428. https://doi.org/10.1126/science.aan6877

Lee, K. J., Tilling, K., Cornish, R. P., Little, R. J., Bell, M. L., Goetghebeur, E., Hogan, J. W., & Carpenter, J. R. (2020). *Framework for the Treatment And Reporting of Missing data in Observational Studies: The TARMOS framework*. http://arxiv.org/abs/2004.14066

Madley-Dowd, P., Hughes, R., Tilling, K., & Heron, J. (2019). The proportion of missing data should not be used to guide decisions on multiple imputation. *Journal of Clinical Epidemiology*, *110*, 63–73. https://doi.org/https://doi.org/10.1016/j.jclinepi.2019.02.016

Nowicki, S., & Strickland, B. R. (1973). A locus of control scale for children. *Journal of Consulting and Clinical Psychology*, *40*(1), 148.

Royston, P. (2005). Multiple imputation of missing values: update of ice. *The Stata Journal*, *5*(4), 527–536.

Royston, P. (2007). Multiple imputation of missing values: further update of ice, with an emphasis on interval censoring. *The Stata Journal*, *7*(4), 445–464.

Sterne, J. A. C., White, I. R., Carlin, J. B., Spratt, M., Royston, P., Kenward, M. G., Wood, A. M., & Carpenter, J. R. (2009). Multiple imputation for missing data in epidemiological and clinical research: potential and pitfalls. *Bmj*, *338*, b2393.

Thomas, A., & Chess, S. (1977). *Temperament and development.* Brunner/Mazel.

VanderWeele, T. J., & Hernán, M. A. (2012). Results on differential and dependent measurement error of the exposure and the outcome using signed directed acyclic graphs. *American Journal of Epidemiology*, *175*(12), 1303–1310.

Warrington, N. M., Hwang, L.-D., Nivard, M. G., & Evans, D. M. (2021). Estimating direct and indirect genetic effects on offspring phenotypes using genome-wide summary results data. *Nature Communications*, *12*(1), 5420. https://doi.org/10.1038/s41467-021-25723-z

Wechsler, D., Golombok, S., & Rust, J. (1992). WISC-III UK. *Sidcup, Kent: The Psychological Corporation*.
